# Side-Chain Free Semiconducting Polymer for High-Performance n‑Type Organic Electrochemical Transistors

**DOI:** 10.1021/jacs.5c19399

**Published:** 2026-02-26

**Authors:** Yuyun Yao, Mustafeez Bashir Shah, Wanpeng Lu, Xian’e Li, Rushil Vasant, Zeinab Hamid, Keren Ai, Junfu Tian, Maryam Alsufyani, Jonathan Rawle, Malina Gaşpar, Qingpei Wan, Rachael Found, Wesley Chen, Tomaž Kotnik, Thuc-Quyen Nguyen, Achilleas Savva, James Durrant, Iain McCulloch

**Affiliations:** † Chemistry Research Laboratory, 6396 University of Oxford, 12 Mansfield Road, Oxford OX1 3TA, U.K.; ‡ Andlinger Center for Energy and the Environment and Department of Electrical and Computer Engineering, 629745 Princeton University, Princeton, New Jersey 08544, United States; § Department of Microelectronics, Faculty of Electrical Engineering, Mathematics and Computer Science, 2860 Delft University of Technology, Mekelweg 4, Delft 2628 CD, The Netherlands; ∥ Center for Polymers and Organic Solids, University of California at Santa Barbara, Santa Barbara, California 93117 United States; ⊥ Laboratory of Organic Electronics, Department of Science and Technology (ITN), Linköping University, Norrköping SE- 60174, Sweden; # Department of Chemistry, 4615 Imperial College London, London W12 0BZ, U.K.; ∇ Diamond Light Source, Harwell Science Campus, Oxfordshire OX11 0DE, U.K.; ○ Department of Chemistry, Massachusetts Institute of Technology, 77 Massachusetts Avenue, Cambridge, Massachusetts 02139, United States

## Abstract

The development of organic electrochemical transistors (OECTs) critically depends on the design and characterization of mixed-conducting, high-performance conjugated polymers (CPs) as channel materials, particularly for n-type OECTs. In this study, we present a novel strategy to enhance the OECT performance of a semiconducting polymer film via a postdeposition ester pyrolysis of thermally cleavable side chains, thus facilitating ion incorporation and transport within the bulk. Our approach relies on the synthesis of a high glass-transition, rigid-rod polymer, able to withstand the pyrolysis temperature without deformation and maintain the voids formed from the pyrolysis reaction which removes the thermally cleavable ester side chains. After side-chain cleavage, the resulting film exhibits increased porosity, hydrophilicity, and crystallinity. By creating bulk porosity in thin films via this approach, ion diffusion is enhanced, resulting in a superior μC* figure of merit up to 158.85 F cm^–1^ V^–1^ s^–1^, and a corresponding increase in normalized transconductance (31.67 S cm^–1^). In addition, the device switching speed and long-term stability are also observed to increase, further demonstrating the benefit of nanoscale porosity for mixed conductivity semiconductors.

## Graphical abstract

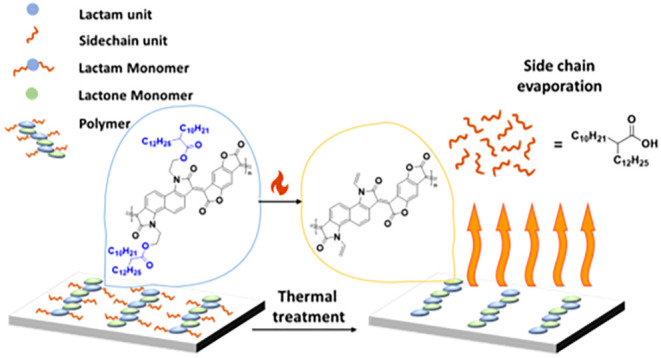

## Introduction

Organic mixed ionic-electronic conductors (OMIECs) are a class of materials capable of transporting both ions and electrical charges, making them ideal channel materials for organic electrochemical transistors (OECTs).^1–3^ OECTs have been applied in various bioelectronic devices, including biosensors,^4^ metabolite sensors,^5^ and electrophysiological recorders.^6^ Under the operating mode of a OECT device, ions from the electrolyte penetrate into the polymer film and compensate the injected charge carriers under an applied external bias.^7^ The transduction efficiency of OECTs is reflected in the output signal via transconductance, given $g_{m}\,\;=\;\frac{\mathrm{Wd}}{L}\mu C^{*}\left(V_{g}-V_{th}\right)$.^8^ *g*_m_ is directly dependent on the channel volume, the width (*W*), length (*L*) and thickness­(*d*) and the material property dependent factors, the charge carrier mobility (μ), and volumetric capacitance (C*).^9,10^

The performance of organic semiconductors has been more extensively optimized in p-type devices than their n-type counterparts.^11,12^ Naphthalene diimide (NDI)^13,14^ -based conjugated polymers are among the most extensively studied donor–acceptor (D-A) n-type OMIECs, offering promising n-type OECT performance. These polymers typically incorporate a mixture of glycol and alkyl side chains, where glycol side chains enhance hydrophilicity^15,16^ to allow ion diffusion, while the alkyl side chains facilitate interdigitation and packing, as well as contribute to solubility, enabling the synthesis of high-molecular-weight polymers.^17,18^ However, the presence of hydrophilic glycol side chains can disrupt structural order, due to excessive swelling from the incorporation of hydrated ions, consequently resulting in lower charge mobility.^19–21^ Additionally, the localized distribution of the LUMO energy levels on the NDI unit contribute to their lower carrier mobility, and their relatively shallow LUMO energy levels render them susceptible to oxidative degradation, limiting long-term stability.^22–24^ Ladder-type polymers, which do not contain side chains, and have deep LUMO energy levels, have emerged as an attractive alternative.^25,26^ Among them, poly­(benzimidazobenzophenanthroline) (BBL) has been shown to exhibit both excellent charge transport and good device stability.^12,27–29^ Without side chains, BBL can accommodate a high density of ionic species on operation arising from its high polaron density, resulting in high volumetric capacitance.^29,30^ The ionic uptake of analogous linear polymers has been lower in comparison, which in part can be attributed to the high density of alkyl side chains and their corresponding hydrophobicity.^29^

To address this limitation, while leveraging the advantages of ladder-type polymers, we explored an alternative design concept to facilitate ion diffusion through the creation of nanopores within the bulk. This is achieved via an in situ side-chain ester pyrolysis cleavage reaction, which can create percolating channels for ion diffusion, as illustrated in Figure 1a, which do not collapse at the pyrolysis temperature employed for the side-chain cleavage reaction. To ensure the integrity of the pores during the pyrolysis, a fully fused, rigid backbone with electron-deficient lactam groups, previously demonstrated to have high electron mobility in an organic field effect transistor, was chosen.^31^ Elimination of aliphatic side chains also reduces the bulk hydrophobicity, improving ion incorporation, and hence further increases the volumetric capacitance. Previous work has shown it is possible to thermally deprotect BOC functionalized DPP polymers, with the deprotected analogues possessing promising transport properties.^32–34^ More recently, it has been shown that this methodology can also be used in situ to afford mixed transport semiconductors for OECT devices.^35^ However, in all cases, post reaction porosity was not reported, likely due to the glass transition (*T*_g_) of the polymer being lower than the deprotection temperature. In this work, high *T*_g_, thermally cleavable lactam-based polymers have been designed which feature an intrinsically planar and rigid backbone enabling high electron mobility and air stability.^36^ Furthermore, the side chains can be removed by an ester pyrolysis reaction to the constituent alkyl carboxylic acid and a backbone olefin through a cis-elimination reaction at high temperatures, leading to the generation of internal nanoporosity and enhanced backbone planarity and rigidity. This reduced hydrophobicity can be exploited in mixed transport applications, facilitating ion diffusion into the bulk and increasing volumetric capacitance and switching speed. The chemical structure of this ladder-type polymer, poly alkyl-naphthalene-lactone­(paNL), is shown in Figure 1b. Upon thermal cleavage, the ester groups undergo intramolecular pyrolysis via 1,5-proton migration, yielding a poly alkene-naphthalene-lactone (peNL) while releasing alkyl carboxylic acid as a byproduct.^37^ The removal of aliphatic side chains introduces intrinsic porosity into the film, an approach previously demonstrated to improve OECT performance, for example by the breath-figure patterning method^38,39^ and solvent-induced phase separation (SIPS).^40^ The porosity introduced through side-chain cleavage offers the potential for an interconnected network that facilitates ion penetration and accelerates volumetric charging, thereby enhancing device performance. In addition, the thermal cleavage generates mesopores with uniform and controllable size of about 2–4 nm, corresponding to the side-chain length. Moreover, this chemical design provides a novel synthetic route for generating alkene side groups on conjugated aromatic polymer backbones.

**Figure 1:**
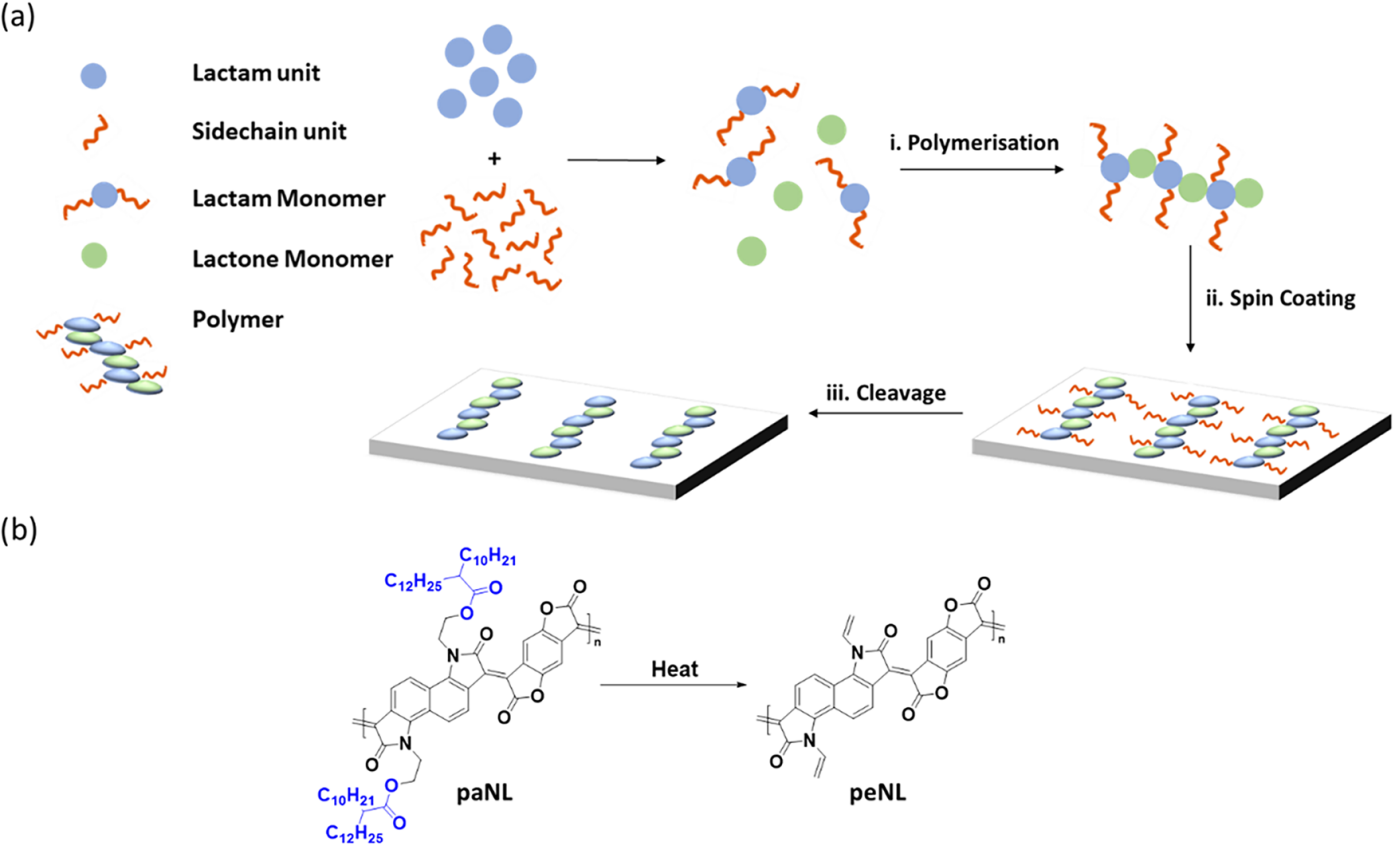
Schematic illustration of synthetic properties and thermal cleavage process: (a) General thermal treatment procedure for making porous films. (b) Chemical structure and thermal cleavage conditions for paNL and peNL.

## Results and Discussion

The electron-deficient conjugated polymer paNL was designed and synthesized, as shown in Figure 1b, based on the aldol condensation of bis-isatin and benzodifurandione functional groups to form all-fused electron-deficient lactam and lactone rings. The synthetic details are shown in Figures S1–S3. First, naphthalene-bis-isatin was prepared through a Martinet dioxindole condensation reaction, following established literature procedures.^41,42^ The core was then subjected to an *n*-alkylation reaction with bromine-terminated, ester-functionalized side chains, resulting in a naphthalene-bis-isatin monomer. Second, the benzodifuranone was synthesized via a nucleophilic addition of 1,4-benzoquinone by ethyl cyanoacetate, followed by hydrolysis. The resulting diacid intermediate was subjected to intramolecular esterification to obtain the benzodifurandione monomer (Figures S4 and S5).^43^ paNL was subsequently synthesized via a metal-free aldol condensation.^36,44^ P-toluenesulfonic acid (PTSA) served as the acid catalyst to drive the condensation polymerization between the enolic bis-lactone and electrophilic bis-isatin, producing water as the only byproduct.^36^ The polymer paNL was purified through Soxhlet extraction via methanol, ethyl acetate, acetone, hexane, and chloroform, and its chloroform fraction was collected with a number-average molecular weight (Mn) of 15.8 kg/mol, weight-average molecular weight (Mw) of 32.6 kg/mol, and PDI 2.06. The detailed synthesis and characterization of paNL including 1H NMR and GPC trace are provided in Figures S6–S8.

### Thermal Properties and Energy Level Investigations

The thermal stability and appropriate cleavage temperature of paNL were evaluated using thermogravimetric analysis (TGA) under a nitrogen environment. Optimization experiments were conducted at various temperatures at 250, 270, and 300 °C to determine an appropriate decomposition temperature for the removal of alkyl side chains (Figures S9–S12). Among these conditions, 300 °C was identified as the most effective temperature, enabling efficient side-chain cleavage while retaining the integrity of the polymer vinyl bond as much as possible. To ensure complete removal, the samples were held at 300 °C for 12 h, as depicted in Figure 2a. The plotted curve of weight percentage versus holding time shows a pronounced weight loss followed by a gradual plateau after approximately 400 min, indicating that all volatile mass components were fully removed at this temperature. In Figure 2a, paNL exhibits an overall mass loss of approximately 60% at 300 °C, which corresponds well with the theoretical mass ratio of the cleavable side chains. Moreover, upon further heating to 350 °C at a rate of 10 °C min^–1^, no additional mass loss was observed between 300 and 350 °C, indicating complete side-chain removal under isothermal conditions. In addition, TGA demonstrates that the cleaved polymer backbones possess good thermal stability, with decomposition temperatures exceeding 350 °C. After heat treatment, the polymer became insoluble in water and all tested organic solvents (e.g., chloroform, *N,N*-dimethylformamide (DMF), and dimethyl sulfoxide (DMSO)), details described in Figures S13 and S14. The condensed byproducts were collected and identified by ^1^H NMR and high-resolution mass spectrometry (HRMS) in Figure S15, confirming that the majority of the byproducts were comprised mainly of the expected 2-decyltetradecanoic acid, along with a minor fraction of decomposed fragments. Differential scanning calorimetry (DSC) was performed on peNL over a temperature range of −78 to 300 °C to investigate thermal transitions of the conjugated polymer (Figure S16). No distinct melting endotherm, crystallization exotherm, or glass transition was observed during either the heating or cooling cycles.

**Figure 2:**
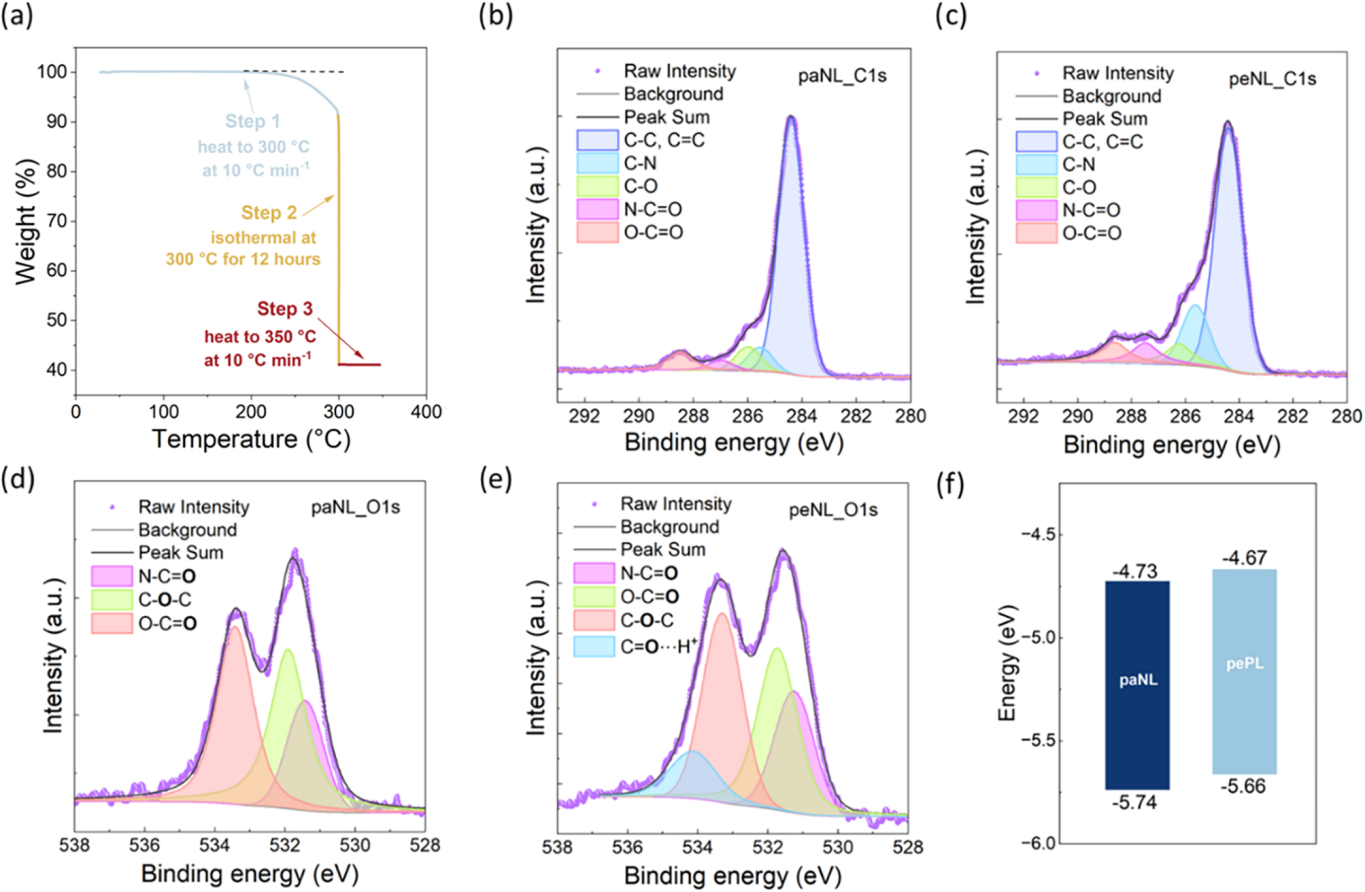
Thermal and structural properties of paNL and peNL: (a) Thermogravimetric analysis (TGA) of the cleavage process. (b) XPS C 1s spectra of paNL and (c) peNL. (d) XPS O 1s spectra of paNL and (e) peNL. (f) Energy levels for paNL and peNL; The value at the bottom of the bar corresponds to the ionization potential (IP) measured by ultraviolet photoelectron spectroscopy (UPS) and the value at the top of the bar corresponds to the electron affinity (EA) estimated from the optical gap calculated using the onset of absorption spectra (*E*_opt.gap_ = 1240/λ_onset_) and IP.

X-ray photoelectron spectroscopy (XPS) analysis was also performed to evaluate the degree of thermal cleavage on thin films. Thermal treatments were carried out at 260 °C for 1 h, 300 °C for 30 min, and 300 °C for 1 h to optimize the extent of side-chain cleavage in paNL films. The cleavage was evaluated by monitoring the C 1s peak intensity relative to the N 1s peak in XPS spectra (Figure S17). A significant reduction in the C 1s and O 1s peak intensities, corrected using atomic sensitivity factors and normalized to the constant N 1s peak, was observed only in the peNL film heated at 300 °C for 1 h, indicating effective removal of the side chains under these conditions (Figure S18). The fitted C 1s spectra of paNL and peNL exhibit five components: C–C/C=C, C–N, C–O, N–C=O, and O–C=O. (Figure 2b,2c, Table S1) The elemental ratios of these carbon species match well with the theoretical structure of paNL (C–N: C–O: N–C=O: O–C=O = 2:2:1:2) and shift accordingly after thermal cleavage, which are consistent with the theoretical ratios for peNL (2:1:1:1). Moreover, the O 1s spectra further supports the chemical structures for paNL and peNL (Figure 2d,2e). paNL displays a 1:2:2 ratio among carbonyl oxygen in an amide group (N–C=O), carbonyl oxygen in a carboxyl group (O–C=O), and ether oxygen (C–O–C).^45^ Upon thermal cleavage, a new high-binding-energy peak emerges, similar to those observed in n-type lactone-based conducting polymers,^46,47^ which is attributed to hydrogen-bonded amide carbonyls (N–C=O···H). The combined intensities of the hydrogen-bonded and non-hydrogen-bonded N–C=O oxygen peaks, relative to those of O–C=O and C–O–C, are approximately in a 1:1:1 ratio, which is consistent with the theoretical structure of peNL. Importantly, the overall spectral profiles for each element remain largely consistent, suggesting that the polymer backbone is largely preserved.

The UV–vis-NIR absorption spectra of polymer thin films are presented in Figure S19. Both polymers exhibited absorption maxima of 949 nm (paNL) and 945 nm (peNL), respectively, and with broad absorption up to 1200 nm, corresponding to a narrow band gap of approximately 1.0 eV. The UV spectra of both polymers displayed similar λ_max_ values and onset potentials, consistent with the polymer backbone conjugation remaining intact after the decomposition of the ester side chains. To estimate the energy level of the frontier orbitals, a Density Functional Theory calculation was performed using Gaussian09 with the B3LYP/def2SVP basis set. To simplify the simulation, the long solubilizing side chain was reduced to a methyl ester and the polymer backbone was shortened to a dimer fragment (Figures S20 and S21).^48^ Removal of the side chain did not significantly influence the energy levels of the simulated frontier orbitals. In both cases, the dimeric segments possessed nearly identical HOMO and LUMO levels of −5.7 and −4.0 eV, respectively. In addition, the distribution of molecular orbitals remained unaltered upon removal of the side chain, accounting for the similarity in the energy levels for both effective dimeric segments. Ionization potentials (IPs) for the two polymers were determined using ultraviolet photoelectron spectroscopy (UPS) and photoelectron spectroscopy in air (PESA) as shown in Figure S22 and electron affinities (EAs) were calculated by subtracting the optical band gap from the IP Anchorvalues. These results are summarized in Figure 2f. paNL exhibited a large ionization potential of approximately 5.7 eV, arising from the electron withdrawing lactam and lactone groups within the backbone. Upon thermal treatment, the ionization potential of peNL remained nearly unchanged, indicating that the side-chain cleavage had minimal influence on the electronic structure of the conjugated backbone. The polymers show large EAs around 4.7 eV, which all exceed the reduction potentials of oxygen E^0^ O_2_/O_2_^•–^ = −0.33 *V*_SHE_ (−4.11 eV vs vacuum) and the reduction potential of water E^0^ H_2_O/H_2_ = −0.41 *V*_SHE_ (−4.05 eV vs vacuum).^23,49^ These deep LUMO levels enable the polymer to resist oxidation of negative polarons in operation.^50^ In addition, the EAs were determined from the reduction onset potentials relative to the ferrocene/ferrocenium (Fc/Fc+) redox couple as measured by organic cyclic voltammetry (CV). The onset reduction potentials for paNL and peNL were calibrated against the Fc/Fc+ redox couple, yielding values of −0.05 and 0.1 eV, respectively, as shown in Figure S23. Based on these measurements, the corresponding EAs were calculated to be 4.65 eV for paNL and 4.8 eV for peNL, as summarized in Table S2. The increased EA of peNL under electrochemical conditions is supposed to better solvate and stabilize injected electrons and is expected to be beneficial for the operation of the OECT, as it enables electrochemical doping at lower applied bias.

### In Situ Spectroelectrochemistry (SEC)

The in situ spectroelectrochemical reduction of paNL and peNL films were measured with UV/vis-NIR under applied negative bias between 0 V and −0.7 V (Figure 3a,3b), in order to avoid double reduction. At voltages above 0 V, peNL exhibited an increasingly pronounced absorption band peaking at 1300 nm, attributed to electron polarons, which corelates with its lower reduction onset observed by aqueous CV measurements (Figure 3d,3e), which also demonstrate a more reversible behavior after cleavage. In contrast, paNL required a more negative potential (−0.2 V) to display a comparable electron polaron absorption at 1300 nm, indicating a higher reduction onset, in agreement with the CV results. Figure 3c presents a comparison of the SEC spectra kinetics for electron polaron absorption at 1300 nm and its corresponding bleach signal at 950 nm, associated with the reduction in π–π* absorption from the polymer backbone.^51^ Interestingly, both films reached a similar amplitude of the bleach signal (950 nm) at −0.7 V. However, peNL displayed higher electron polaron absorption than did paNL, consistent with the higher current revealed in the CV plots with the same electrolyte (0.1 M NaCl). This indicates that at the potential where π–π* absorption reduction is equivalent, peNL accommodates more photochemically induced electrons than paNL per mol without any degradation, evidenced by the reversible SEC spectra (Figure S24). Furthermore, the isosbestic point of peNL spectra blue-shifted from around 1100 to 1075 nm after thermal treatment. This can be attributed to changes in the electronic structure or morphology of the polymers induced by the removal of the side chains. Under the application of a reverse bias starting from −0.7 to 0 V, the peak at 950 nm gradually reappeared and the polaron absorption diminished. The reverse SEC spectra for both polymers exhibited similar shapes and magnitudes compared to their corresponding forward SEC spectra. This observation confirms that both polymers demonstrated excellent stability under the application of an external bias.

**Figure 3:**
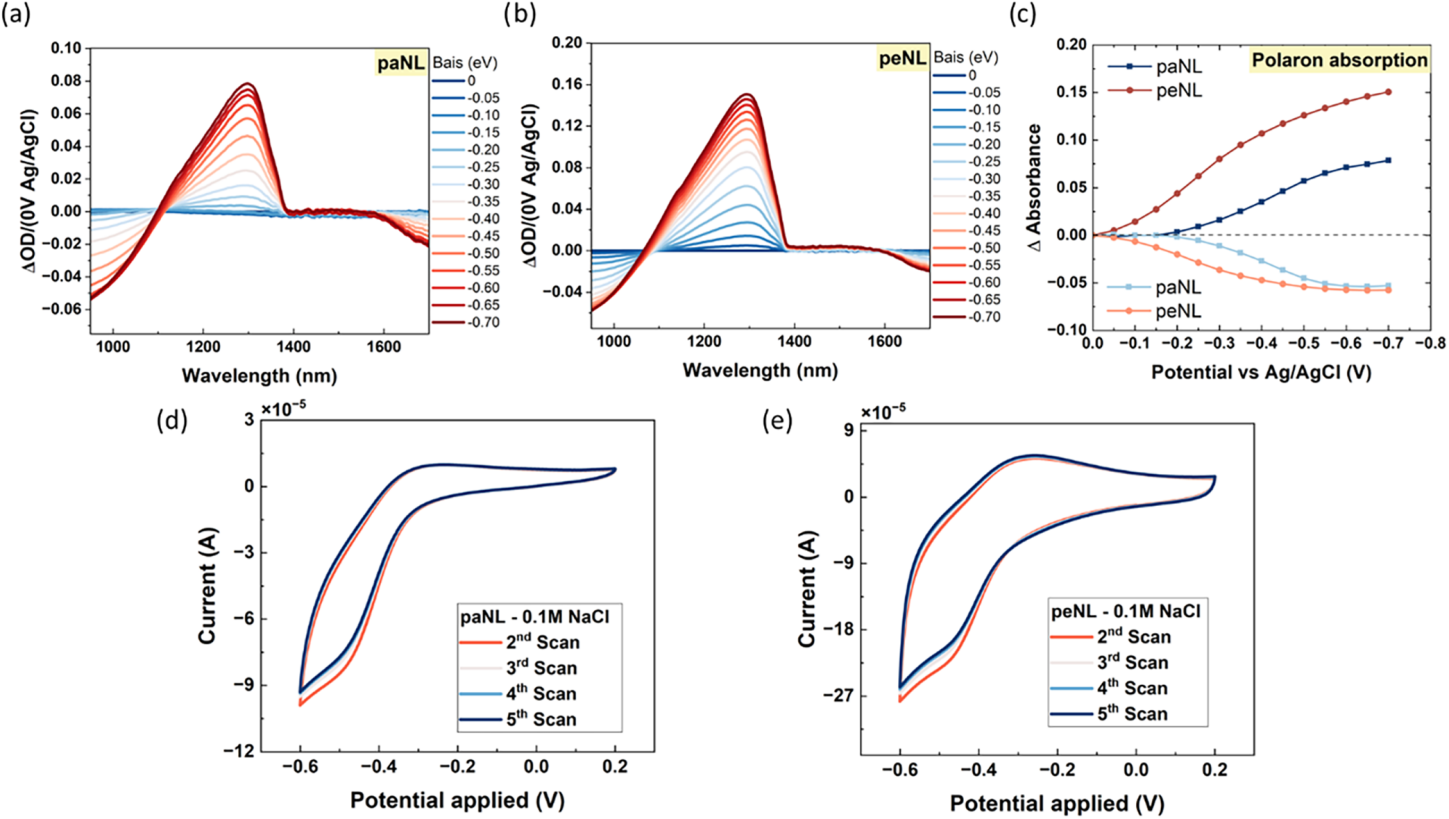
Spectroelectrochemistry (SEC) of paNL and peNL: Potential dependent UV/vis-NIR absorption spectra (900 to 1700 nm) of (a) paNL, (b) peNL, and (c) absolute changes in polaron absorption and π–π* absorption versus applied bias of paNL and peNL. Cyclic voltammetry (CV) in 0.1 M NaCl of (d) paNL and (e) peNL.

### Porosity and Hydrophilicity

Brunauer–Emmett–Teller (BET) measurements were conducted to evaluate the porosity of the polymer powder before and after the thermal cleaving process. As shown in Figure 4a, the nitrogen uptake in paNL at 77K is below 1 cm^3·^g^–1^, while after the thermal cleavage event at 300 °C, peNL showed an 8-fold increase in nitrogen adsorption. The BET surface areas for paNL and peNL are calculated to be 4.2 and 18.1 m^2·^g^–1^, respectively. This increase in the nitrogen uptake and calculated surface area suggests the introduction of porosity into the structure, arising from the thermal removal of alkyl side chains. The magnitude of this effect may be compromised however by the high temperature anneal and concomitant pore filling by backbone reptation. In context, PIM polymers have been shown to exhibit much higher porosity; however, peNL has been designed to additionally promote charge carrier mobility through intermolecular π-stacking, which reduces the free volume obtainable. The size of the pores mainly ranges from 2 to 4 nm as calculated by Barrett–Joyner–Halenda (BJH) method (Figure 4b).^52,53^ To further investigate the changes in hydrophilicity as a result of the thermal cleavage, water vapor sorption isotherms were conducted, with the results presented in Figure 4c, revealing both water adsorption and desorption in paNL, indicating its inherent hydrophilicity at about 55 cc·g^–1^ at 298 K. After thermal treatment at 300 °C, peNL exhibited twice the water uptake compared to paNL at atmospheric pressure, exceeding 120 cc·g^–1^. These isotherm measurements confirm that the removal of hydrophobic alkyl side chains creates the desired nanoporosity as well as increases the overall polarity of the polymer, with a corresponding increase in hydrophilicity. Both TEM and cryo-TEM were employed to investigate the porosity, with samples prepared by various methods and staining techniques. However, these techniques were unsuccessful in resolving the pores, likely due to their small size and the low contrast associated with the side-chain-length-scale porosity.

**Figure 4:**
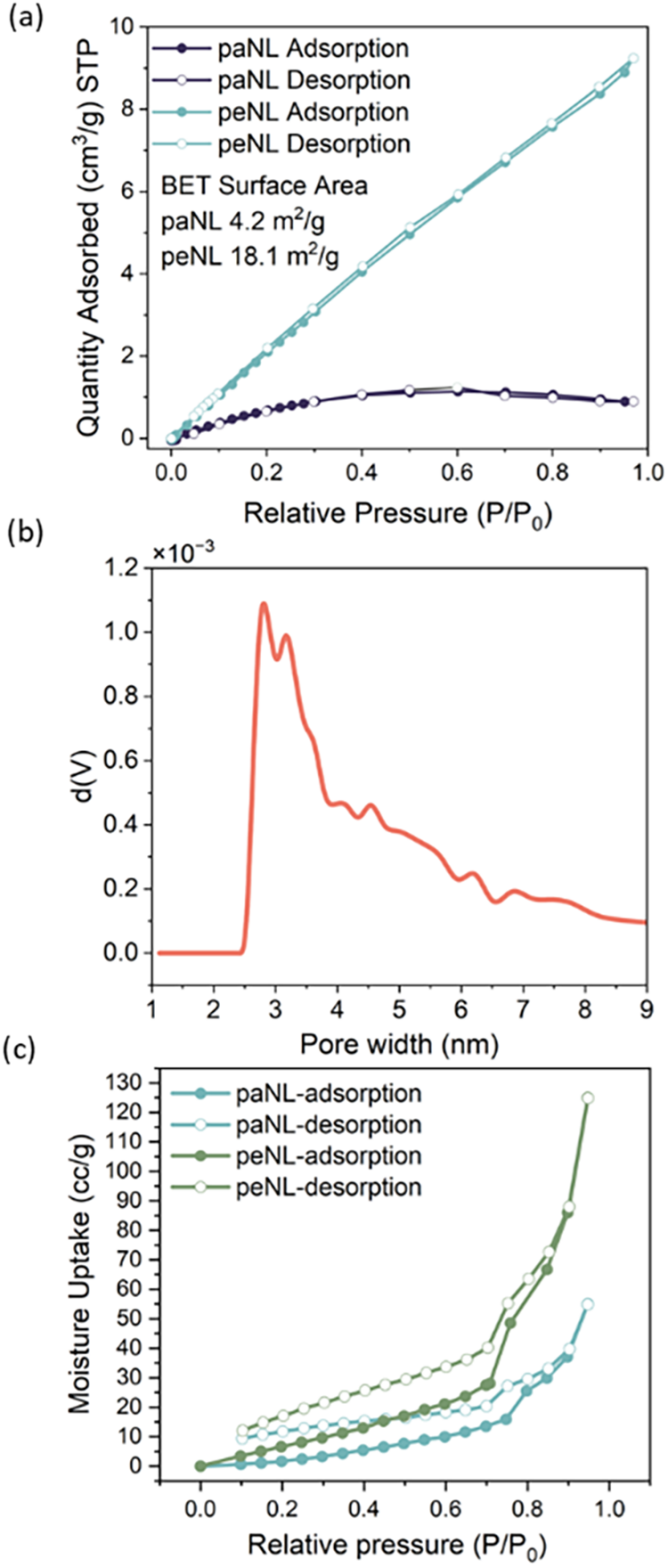
Investigation of material porosity and pore hydrophilicity for paNL and peNL: (a) Nitrogen isotherm of paNL and peNL at 77K and calculated BET surface area. (b) Pore size distribution for peNL calculated by Barrett–Joyner–Halenda (BJH) method. (c) Water isotherm measured for paNL and peNL at 298 K.

The hydrophilicity and swelling behavior of paNL and peNL thin films were examined via static water contact angle measurement and liquid atomic force microscopy (AFM). Contact angle measurements (Figure S25, Table S3) revealed a contact angle of 96° for paNL, indicating a hydrophobic surface that limits ion penetration and favors ion accumulation at the film and electrolyte interface, forming a double-layer capacitance.^54^ In contrast, peNL exhibited a reduced contact angle of 72°, consistent with enhanced hydrophilicity following side-chain cleavage. This increase in hydrophilicity results in the enhanced volumetric capacitance of peNL. To further evaluate the influence of hydrophilicity on swelling under conditions relevant to the operation of the OECT, AFM film thickness measurements were conducted in air and in aqueous fluid (0.1 M NaCl) as shown in Figure S26. From these measurements, paNL displayed an 11% increase in thickness (from 86.8 to 96.5 nm), while peNL exhibited an 18% increase (from 79.3 to 93.6 nm) when transitioning from the dry to the swollen state. These results align with the discussion above, indicating the improved hydrophilicity and swelling ability of the bulk peNL thin film.

### Film Crystallinity and Morphology

Grazing Incidence Wide Angle X-ray Scattering (GIWAXS) was performed to evaluate the microstructure for paNL and peNL thin films and observe changes arising from the thermal cleavage process and subsequently relate morphology differences to the performance of the OECT. paNL displayed an isotropic ring scattering pattern (Figure 5a) centered around 1.5 Å^–1^ (real space distance 4.2 Å) and diminished degrees of lamellar scattering, indicating a broad distribution of crystallite orientations.^44,55,56^ For paNL, scattering from the backbone (1.5 Å^–1^) is mainly observed in-plane, accompanied by an isotropic ring centered at 1.5 and 1.8 Å^–1^, respectively, indicating no preferred orientation. Whereas for peNL, the π-π stacking (010) scattering at 1.8 Å^–1^ (π-π stacking distance 3.5 Å) is now clearly observed in-plane and backbone (1.5 Å^–1^, real space distance 4.2 Å) out-of-plane, corresponding to a dominant edge-on orientation. Furthermore, distinct lamellar scattering (100) and (200) are identified in the linecuts of the out-of-plane direction at 0.5 Å^–1^ (real space distance 12.9 Å) and 1.0 Å^–1^ (lamellar spacing 6.5 Å), respectively (Figure 5b). This variance suggests a significant increase in crystallinity of peNL as a result of the thermal cleavage process. However, this difference in crystallinity is not observed by DSC, likely due to the sensitivity of DSC to larger scale transitions, limiting its ability to observe melting of either short-/medium-range ordered structures or low crystalline fractions. This structural transformation does not noticeably affect the surface morphology of the films, as evidenced by AFM topography images (Figure 5c). The AFM measurements reveal comparable surface features and root-mean-square (RMS) roughness values for paNL (1.04 nm) and peNL (0.87 nm), despite the enhanced molecular ordering in peNL observed by GIWAXS. This contrasts with previous reports on thermocleavable small molecules, where side-chain removal resulted in significant morphological restructuring.^54^

**Figure 5:**
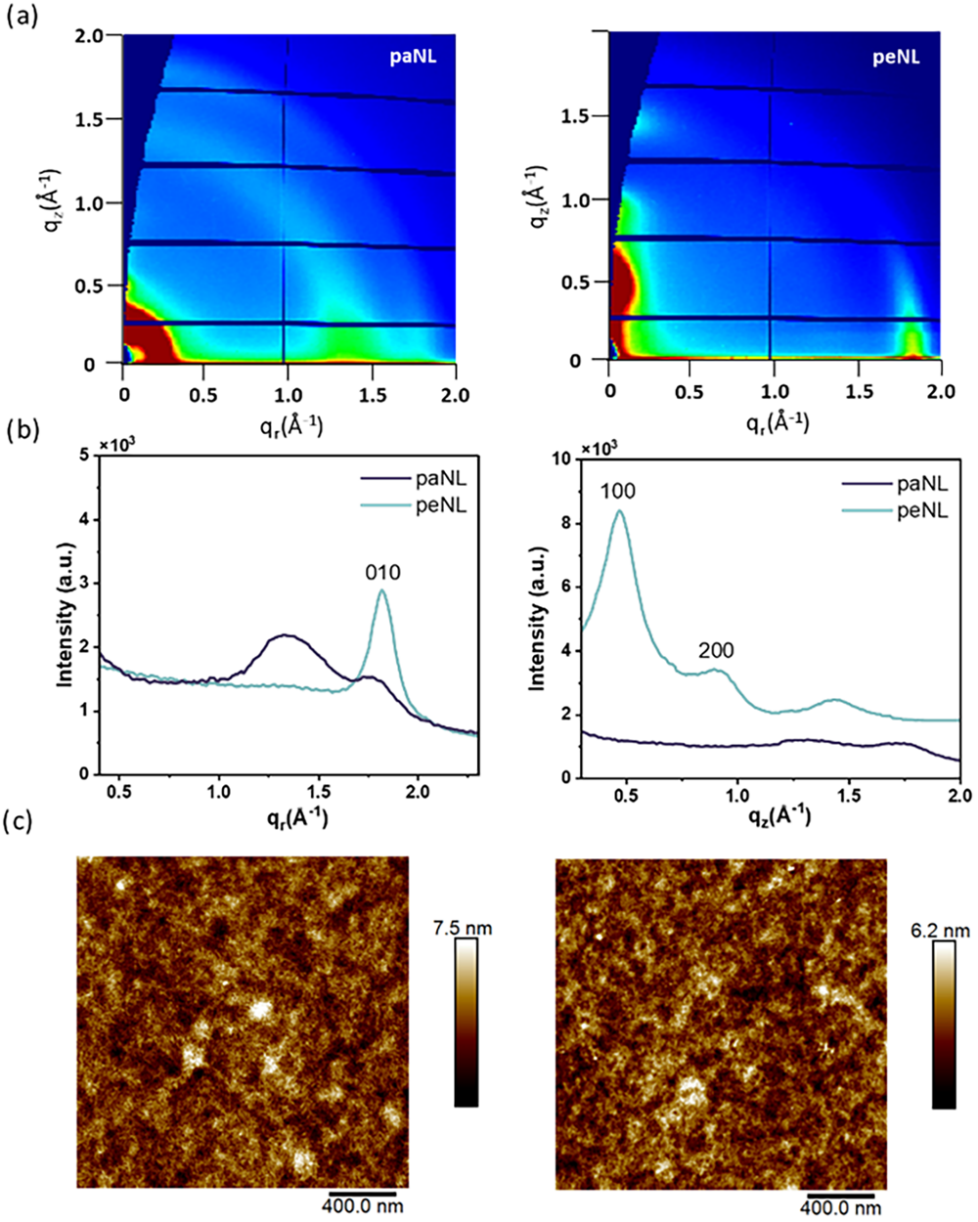
Structural characterization of the representative paNL and peNL thin films: (a) Two-dimensional grazing incidence X-ray scattering (GIWAXS) image of paNL and peNL. (b) In-plane and out-of-plane 1D linecuts of paNL and peNL’s 2D GIWAXS patterns. (c) AFM topography images of paNL and peNL.

### Organic Electrochemical Transistors

OECT devices were fabricated by depositing paNL onto silicon wafer substrates patterned with gold electrodes (Figure S27). The resulting paNL films were annealed in an anaerobic environment at 300 °C for 1 h to remove the side chains, yielding peNL. The peNL films were subsequently immersed in a 0.1 M NaCl electrolyte and operated using a top gate Ag/AgCl electrode. As shown in Figure 6a–6c, both paNL and peNL show an increase in the drain current (*I*_D_) upon the increase of the gate voltage (*V*_G_), indicative of an accumulation mode OECT. The corresponding transfer characteristics for both polymers plotted on a logarithmic current scale are provided in Figure S28. In addition, the OECT performance of peNL measured on the same interdigitated electrode geometry was benchmarked against that of an analogous glycolated polymer, P50, previously reported in the literature (Figure S29 and Table S4).^50^ Under identical device geometry, peNL exhibits a superior performance compared to its glycolated analogue. Importantly, the threshold voltage for peNL OECTs (0.21 V) is lowered by ∼0.2 V as compared to paNL (0.39 V) (Figure S30), consistent with the aqueous CV results. Consequently, the gate voltage corresponding to maximum transconductance (i.e., 30 S cm^–1^) occurs at a lower value of 0.4 V for peNL OECTs in contrast to 0.6 V (i.e., 11.89 S cm^‑1^) for paNL OECTs with the same geometry. These performance metrics highlight a shift in the operation region of the polymer from a high voltage for paNL OECTs (*V*_G_ = 0.4–0.65 V) to a lower voltage regime for peNL OECTs (*V*_G_ = 0.2–0.4 V). This shift in the operation to lower voltages can arise from more mobile ion diffusion due to the porosity induced by cleavage of the side chains (as shown in Figure 4a), lowering the drift voltage required for compensating the electronic charge. The lower voltage operation region of the porous OECT channels is a feature that can be particularly advantageous for a number of bioelectronic applications, such as organic electrochemical neurons^57^ where low-voltage operation is important.

**Figure 6:**
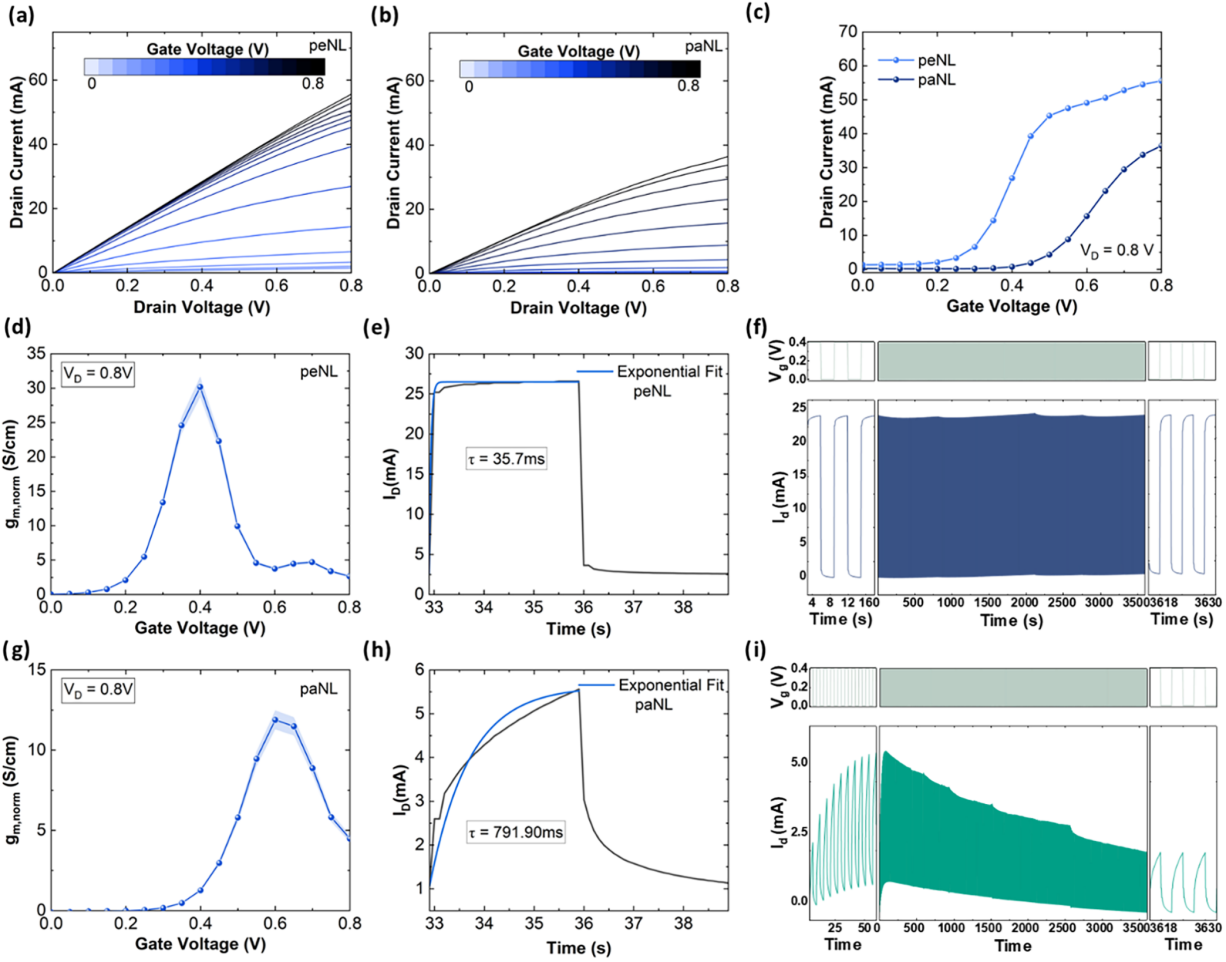
OECT device performance of peNL and paNL: Representative output characteristic curves for (a) peNL and (b) paNL. (c) Standard transfer curves for paNL and peNL. (d) Representative geometry normalized transconductance vs gate voltage curve for peNL using the dimensions of OECTs – i.e., *W*/*L* = 3750. (e) Transient response to a gate voltage pulse of 0.4 V 3 s long and (f) operational stability of the devices pulsed with a 0.4 V gate pulse 3 s in duration for peNL OECT polymer channels. (g) Representative geometry normalized transconductance vs gate voltage curve, (h) transient response, and (i) operational stability of the devices pulsed for paNL OECT polymer channels. The thicknesses of the channels were measured at 32 and 22 nm for paNL and peNL, respectively.

By using this interdigitated electrode device, a high transconductance of 250 mS at *V*_D_ = 0.8 V was achieved in peNL, representing one of the highest values reported to date for n-type OECTs (Table S5). To eliminate any channel geometry effects, the normalized transconductance of peNL was calculated to be approximately 30 S cm^–1^, nearly a 3-fold increase compared to paNL (11.89 S cm^–1^). To further probe the mixed conduction properties of the polymer after cleavage, μC* was extracted from the transconductance plots (Figure 6d,6g). Consistent with the geometry normalized transconductance, a significant improvement is observed in the μC* of peNL, increasing from 56.61 F cm^–1^ V^–1^ s^–1^ for paNL at *V*_G_ = 0.6 V to 158.85 F cm^–1^ V^–1^ s^–1^ for peNL at *V*_G_ = 0.4 V. To elucidate the origin of this increased μC* and decouple the effects of the volumetric capacitance and mobility, the electrochemical impedance spectroscopy measurements were performed and the corresponding volumetric capacitance were calculated as shown in Figure S31. The C* at the corresponding gate voltage of maximum transconductance is approximately double for peNL than paNL (i.e., 227 vs 115 F/cm respectively), which can be correlated with the improved penetration of ions within the bulk of the polymer after cleavage of side chains. The electron mobility values, extracted from the C* of paNL and peNL OECT channels at the corresponding gate voltage of maximum transconductance, were 0.49 and 0.60 cm^2^ V^–1^ s^–1^, respectively. This improvement in mobility might result from the slightly enhanced backbone planarity via intramolecular hydrogen bonding between alkene protons and amide carbonyl oxygens in the lactam unit, as evidenced by XPS analysis.

Furthermore, the improved ionic transport in peNL was corroborated by the response time measurement of the device. As shown in Figure 6e,6h, the switch ON speed for peNL is over 20 times faster compared with that of paNL. This improvement can be attributed to a faster ion diffusion in the peNL device compared to that of paNL. Specifically, the switch ON time of the OECT channels made for peNL was found at 36 ms compared to 792 ms for the OECT channel made with paNL. A summary of all key OECT parameters for both polymers is provided in Table 1. Additionally, peNL demonstrates improved operational stability, maintaining a stable current after 600 continuous switching cycles (Figure 6f,6i). This enhanced stability likely arises from a reduced morphological disruption induced by diffusion of ions in the porous peNL bulk channel. Overall, this work establishes a novel design principle based on thermocleavable ester side chains to enhance OECT performance and suggests a broader applicability of this strategy to other cleavable functional groups, for example, carbonates, as shown in Figure S32.

**Table 1:** Average Polymer OECT Performance of paNL and peNL

polymer	*V*_TH_ [V]^a^	*V*_G_ at *g*_m,max_ [V]^b^	C* at *g*_m,max_ [F/cm^3^]^c^	μC* [F cm^–1^ V^–1^ s^–1^]^d^	μ [cm^2^ V^–1^ s^–1^]^e^	*g*_m_/(*Wd*/*L*) [S cm^–1^]^f^	response time [ms]^g^
paNL	0.39	0.6	115 ± 5	46.9 ± 9.7	0.41 ± 0.08	12.7 ± 1.0	792
peNL	0.21	0.4	227 ± 2	136.2 ± 22.6	0.60 ± 0.13	28.4 ± 5.1	36

a Calculated from the slope of the √ID versus *V*_G_ curve.

b Obtained from the transfer curve of the OECTs.

c Obtained from EIS of microfabricated electrodes.

d Calculated from the slope of *g*_m_ as a function of (*Wd*/*L*) (*V*_TH_ – *V*_G_).

e Calculated from the μC* by using C* extracted from EIS at different *V*_G_.

f Obtained by diving *g*_m_ by the geometrical factor (*Wd*/*L*).

g OECT switch on time obtained from pulsed response time measurements.

## Conclusions

A semiconducting polymer was designed and synthesized to incorporate a thermally cleavable side chain. Conditions were optimized to carry out a postdeposition thermal cleavage reaction on the polymer thin film, which removed the hydrophobic alkyl side chains, resulting in a short alkene functional group on the rigid polymer backbone. Chemical analysis revealed that the side chains could be completely removed at 300 °C over a 1 h period. This thermal cleavage reaction created internal porosity within the bulk of the material as well as an increase in polymer hydrophilicity. Spectroelectrochemistry studies showed that the cleavage process leads to an enhanced polaron formation, while GIWAXS evaluation demonstrated an increased thin film crystallinity after thermal cleavage. Electrochemical transistors were fabricated employing the polymer which revealed an increase in the volumetric capacitance after thermal cleavage, leading to an overall improvement in the μC* product and transconductance. Interestingly, the device response time greatly decreased and the operational stability improved. These results show a new design route toward low-voltage operation of OECT devices with high stability.

## Supplementary Material



## Notes

Additional experimental details, materials, and methods, including photographs of experimental setup are provided in SI (PDF)
