# Correction to “Side-Chain Free Semiconducting Polymer for High-Performance n‑Type Organic Electrochemical Transistors”

**DOI:** 10.1021/jacs.6c17620

**Published:** 2026-09-08

**Authors:** Yuyun Yao, Mustafeez Bashir Shah, Wanpeng Lu, Xian’e Li, Rushil Vasant, Zeinab Hamid, Keren Ai, Junfu Tian, Maryam Alsufyani, Jonathan Rawle, Malina Gaşpar, Qingpei Wan, Rachael Found, Wesley Chen, Tomaž Kotnik, Thuc-Quyen Nguyen, Achilleas Savva, James Durrant, Iain McCulloch

**Acknowledgments correction statement:** NSF Grant number 2404409 should be removed from the Acknowledgments section of the published article because it was added by mistake. All other acknowledgments remain unchanged.

The Acknowledgments section should be corrected to read as below:

