# Supplementary material for: Side-Chain Free Semiconducting Polymer for High-Performance n‑Type Organic Electrochemical Transistors
Source: J Am Chem Soc. 2026 Feb 26;148(9):9494–503. doi: 10.1021/jacs.5c19399 (PMC12983318; doi:10.1021/jacs.5c19399)
Supplement: Supplementary file 1 [file ja5c19399_si_001.pdf]

Supporting Information

Side chain free semiconducting polymer for high-performance n-type organic electrochemical transistors

Yuyun Yao[1], Mustafeez Bashir Shah[3], Wanpeng Lu[1], Xian'e Li[1,5], Rushil Vasant[4], Zeinab Hamid[1], Keren Ai[6], Junfu Tian[1], Maryam Alsufyani[1,8], Jonathan Rawle[7], Malina Gaşpar[3], Qingpei Wan[2], Rachael Found[1], Wesley Chen[4], Tomaž Kotnik[1], Thuc-Quyen Nguyen[4], Achilleas Savva[3], James Durrant[1], Iain McCulloch[1,2*]

1. Chemistry Research Laboratory, University of Oxford, 12 Mansfield Road, Oxford, OX1 3TA, UK.

2. Andlinger Center for Energy and the Environment and Department of Electrical and Computer Engineering, Princeton University, Princeton, NJ, 08544, USA.

3. Department of Microelectronics, Faculty of Electrical Engineering, Mathematics and Computer Science, Delft University of Technology, Mekelweg 4, Delft 2628 CD, Netherland.

4. Center for Polymers and Organic Solids, University of California at Santa Barbara, Santa Barbara, CA 93117 USA.

5. Laboratory of Organic Electronics, Department of Science and Technology (ITN), Linköping University, Norrköping SE- 60174, Sweden.

6. Department of Chemistry, Imperial College London, London W12 0BZ, UK.

7. Diamond Light Source, Harwell Science Campus, Oxfordshire, OX11 0DE, UK.

8. Department of Chemistry, Massachusetts Institute of Technology, 77 Massachusetts Avenue, Cambridge, Massachusetts 02139, United States.

Contents:

A. General procedure 3

B. Synthesis of materials 6

C. Annealing procedure investigations 14

D. Preparation and investigation of peNL 17

E. Film annealing procedure: X-ray photoelectron spectroscopy (XPS) analysis 20

F. Thin film ionisation potential and electron affinity characterisations 22

G. In-situ spectroelectrochemistry (SEC) spectra 26

H. Hydrophilicity and swelling behaviour of paNL and peNL.. 27

I. Supporting Figures for OECT 29

J. References 35

Fig. S1 ~ Fig. S26

Table S1 ~ Table S3

A. General procedure and Synthesis of materials

All starting materials were purchased from commercial companies and used without further purification. 2-decyltetradecan-1-ol (97%, 464503), Jones Reagent (758035), thionyl chloride (for synthesis, 8081540100), diethylketomalonate (95%, D97401) was bought from Sigma Aldrich (www.sigmaaldrich.com). 2-bromoethanol (97%, 106921000) 1,5-Diaminonaphthalene (97%, 10704991), ethyl cyanoacetate (98+%, Thermo Scientific Chemicals), and potassium carbonate (anhydrous, 99%, A16625.0C) were purchased from thermo scientific (www.thermofisher.com). All reactions were carried out using commercially purchased solvents and reagents via a standard Schlenk line under nitrogen protection. Acetone, Dichloromethane, N,N-Dimethylformamide (DMF, anhydrous, 99.8%, MFCD00003284), toluene (anhydrous, 99.8%, MFCD00008512), acetic acid (AcOH, glacial, ReagentPlus®, ≥99%), concentrated HCl (37%, ACS reagent), ammonium hydroxide (28% NH3 in H2O, ≥99.99% trace metals basis), benzoquinone (reagent grade, ≥98%), acetic anhydride(99.5%), and ethanol were purchased from Sigma Aldrich (www.sigmaaldrich.com) and were used as received. 1H NMR and 13C NMR spectra were recorded with Bruker 400 MHz AVANCE III NMR spectrometer equipped with CryoProbe (BrukerBioSpin,Rheinstetten, Germany). Residual proton peaks are 7.26 ppm for CDCl3 and 2.52 ppm for DMSO-d6. High resolution Mass Spectra (HRMS) were performed on an ACQUITY I-Class PLUS UPLC System (Waters, Milford, MA, USA) coupled to an ACQUITY RDa mass spectrometer (Waters, Milford, MA, USA) equipped with an ESI probe, in positive ion mode. The polymer molecular weights (number-average (Mn) and weight-average (Mw) and polydispersity were measured using gel permeation chromatography in Chlorobenzene as the mobile phase at 40 °C. Absorption spectra were recorded on Agilent 1260 Infinity II series instrument by preparing polymer solutions in chlorobenzene at 80 °C. Polymer films were prepared by dissolving 10 mg/mL chloroform solutions which is stirred at 40 °C overnight and filtered through a 0.45 µm PTFE syringe filter onto glass slides. Thin film ultraviolet-visible spectra (UV-Vis) were collected on a Shimadzu UV-1800 spectrometer and photoemission spectroscopy in air (PESA) spectra using a KP Technology APS02 system. Solutions for polymer films were prepared by dissolving the polymer at a 10 mg/mL concentration in chloroform which is stirred at 40 °C overnight and filtered through a 0.45 µm PTFE syringe filter. Spin-coating was performed under dynamic conditions at 1000 rpm with an acceleration of 500 for 1 minute onto glass slides. The EAs were determined from the reduction onset potentials relative to the

ferrocene/ferrocenium (Fc/Fc^+) redox couple, as measured by organic cyclic voltammetry (CV). The CVs were conducted in 0.1 M tetrabutylammonium hexafluorophosphate in acetonitrile solution for both polymers with a scan rate of 100 mV s^{-1}.

Brunauer–Emmett–Teller (BET) measurements: All gases for sorption analysis were supplied by BOC at a purity of ≥99.999%. The polymers were precipitated into methanol following Soxhlet purification (or washed if they were insoluble) as described in the polymer synthesis section, then dried in a vacuum oven at 50 °C overnight. For N2 sorption measurements, between 40 to 70 mg polymer was loaded into an Anton Paar Autosorb iQ-XR gas sorption analyser, where they were heated at 120 °C for 8 h under vacuum in situ before beginning the measurement to thoroughly dry the polymers. The N2 sorption isotherms were measured in a liquid N2 dewar. BET surface areas were calculated from the linear regions of the BET plots between relative pressures of 0.01 and 0.03.[1] The pore size distribution is calculated using the BJH method.[2] For water sorption, 40 to 70 mg polymer was loaded into an Anton Paar Autosorb iQ-XR gas sorption analyser, where they were heated at 120 °C for 8 hours under vacuum before beginning the measurement to thoroughly dry the polymers. The water sorption isotherm was measured at 25 °C in a water bath.

The electrodes for the measurement of volumetric capacitance were fabricated via a photolithography process on 4-inch borosilicate glass wafers (UniversityWafer,Inc.). The wafers were first cleaned using sonication by immersing in in Acetone/Isopropanol bath followed by cleaning in 99% fuming Nitric Acid bath, DI water and a final oxygen plasma cleaning step. The wafers were then put through a dehydration bake of 10 minutes at 140 °C followed by HMDS vapor treatment for 60 seconds. A bilayer of LOR5B/AZ ECL3007 (Microchem) was then spin coated for patterning of the metal electrodes, tracks and contact pads via UV-lithography with the Heidelberg MLA150 followed by development in AZ 726MIF (Microchem) developer solution and deposition of Ti/Au (20nm/200nm) with e-beam evaporator (i.e. Temescal FC-20349). The wafers were soaked overnight in a hot PRS-3000 (J.T.Baker) bath at 80 °C followed by ultrasonic agitation in Acetone/IPA and spin drying. Silanization was performed by immersing the plasma-activated substrate (60 s, 25 W, 0.8 mbar) in a 3% (v/v) of methacryloxypropyl trimethoxysilane solution (A-174, Sigma-Aldrich, UK) prepared in 96% ethanol containing 1% acetic acid for 30 seconds, followed by rinsing with ethanol and baking at 70 °C for 1 hour. Subsequently, a 2 μm thick Parylene C (PaC) layer was deposited using chemical vapor deposition (CVD using the SCS Labcoater -

Speciality Coating Systems, US) followed by spin coating of a 2% (v/v) Micro90 soap solution (Cole-Parmer Essentials) at 1000 rpm as an anti-adhesive layer followed by air drying for 15 minutes. A second PaC layer of 2.5 μm was also deposited to act as a sacrificial layer for the patterning of the polymer. This was followed by another photolithography step with AZ10XT photoresist to pattern areas on the PaC to be removed by a dry etching step. Finally, reactive ion etching (Sentech Etchlab 200) was employed to expose the electrode and contact pad areas followed by dicing into individual chips with a glass cutter (Astellatech Inc.) The chips were then immersed in an Acetone/Isopropanol solution to strip the residual photoresist.

B. Synthesis of materials

1. Side chain synthesis

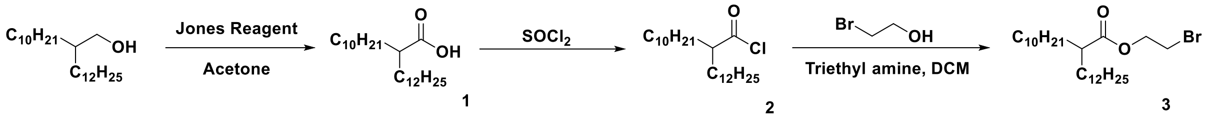

Figure S1. Synthesis of the thermocleavable side chain for monomer M1.

2-decyltetradecanoic acid: The 2-decyltetradecan-1-ol (8 g, 22.6 mmol) was dissolved in acetone (90 mL), cooled to 0 °C for half an hour. The Jones Reagent (42 mL) was then added dropwise to the stirring solution. The mixture was left at room temperature overnight. The resulting solution was quenched with isopropanol until the solution becomes deep green. The mixture was extracted by diethyl ether, and the combined organic phase was collected and washed with 1 M HCl, brine and filtered through Celite. The solvent is removed by rotary evaporator, giving 2-decyltetradecanoic acid (8.13 g, 98%) and carrying out without further purification. ^{1}H NMR (400 MHz, Chloroform-*d*) δ 2.35 (m, 1H), 1.61 (m, 2H), 1.47 (m, 2H), 1.27 (m, 36H), 0.88 (t, J = 6.6 Hz, 6H).

2-decyltetradecanoyl chloride: 2-decyltetradecanoic acid (2 g, 8 mmol) was dissolved in thionyl chloride (5 mL) under nitrogen protection. The mixture was then heat under reflux for 3 hours. The resulting solution was dried in vacuum, and the left brown liquid was collected (2 g, 95%) and used without further purification. ^{1}H NMR (400 MHz, Chloroform-*d*) δ 2.75 (m, 1H), 1.74 (m, 2H), 1.54 (m, 2H), 1.26 (s, 36H), 0.88 (t, J = 6.6 Hz, 6H). ^{13}C NMR (101 MHz, Chloroform-*d*) δ 177.44, 77.33, 77.01, 76.70, 57.25, 32.01, 31.93, 31.91, 31.36, 30.05, 29.72, 29.66, 29.64, 29.62, 29.58, 29.53, 29.40, 29.36, 29.34, 29.32, 26.94, 26.76, 22.70, 22.69, 14.11.

2-bromoethyl 2-decyltetradecanoate: 2-decyltetradecanoyl chloride (2 g, 5.17 mmol) was dissolved in anhydrous DCM (35 mL) and cooled to 0 °C under nitrogen protection. The mixture was added 2-bromoethanol (0.717g, 5.74 mmol) and triethyl amine (0.7 g, 5.74 mmol) and the mixture warmed to room temperature overnight. The resulting mixture was quenched by water. The resulting mixture was then extracted with DCM. The combined organic phase was washed by ammonium chloride, saturated sodium bicarbonate and dried over magnesium sulphate. The crude product was purified by column chromatography (silica gel, DCM : hexane = 3:7) to furnish 2-bromoethyl 2-decyltetradecanoate (2.3 g, 93%) as colourless liquid. ^{1}H NMR

(400 MHz, Chloroform-*d*) δ 4.38 (t, *J* = 6.1 Hz, 2H), 3.50 (t, *J* = 6.1 Hz, 2H), 2.36 (m, 1H), 1.67 – 1.55 (m, 2H), 1.44 (m, 2H), 1.25 (s, 36H), 0.87 (t, *J* = 6.7 Hz, 6H). ^{13}C NMR (101 MHz, Chloroform-*d*) δ 176.09, 63.44, 45.68, 32.44, 31.93, 31.92, 29.68, 29.65, 29.59, 29.54, 29.48, 29.36, 29.34, 28.83, 27.45, 27.26, 22.69, 14.11. HRMS (m/z): (M· +K) calc. ($C_{26}H_{51}BrO_2K$): 513.2704. found: 513.3141.

2. Monomer M1 synthesis

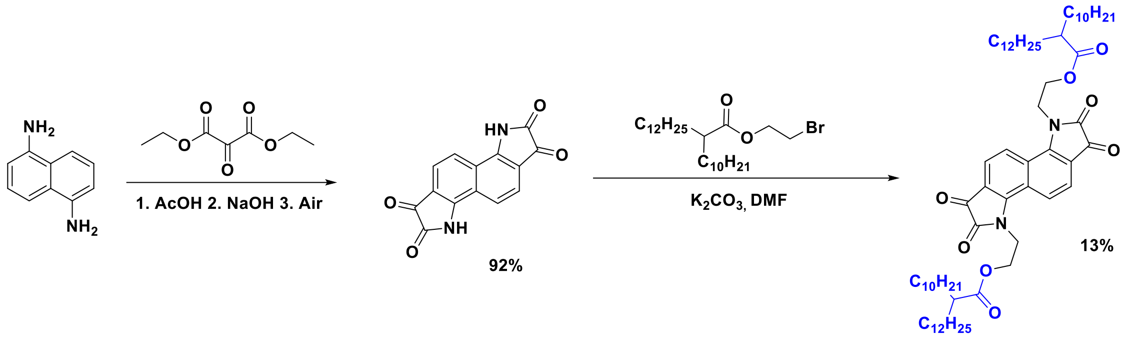

Figure S2. Synthesis of the monomer M1 for polymer paNL.

3,8-dihydroindolo[7,6-g]indole-1,2,6,7-tetraone (Bisisatin): 1,5-Diaminonaphthalene (4 g, 25 mmol) was dissolved in (40 mL) glacial acetic acid and heated to 130°C. The diethylketomalonate (30 mL, 172 mmol) in glacial acetic acid (45 mL) was added dropwise to the resulting solution, and the resulting mixture was heated under reflux overnight. The solvent of the reaction mixture was removed under vacuum and 1M NaOH was added to the resulting solids until complete dissolution to a final pH of (11-12). The resulting solution was heated to 150 °C overnight with sparging air. The resulting mixture was then poured onto ice and 6M HCl was added until pH=0 to give a purple precipitate. The solid was filtered and washed with water to give dark red-purple solids (6 g, 92% yield) and carried out without further purification. ^{1}H NMR (400 MHz, DMSO- *d6*) δ 11.76 (s, 1H), 7.76 (d, *J* = 8.3 Hz, 1H), 7.58 (d, *J* = 8.4 Hz, 1H). ^{13}C NMR (126 MHz, DMSO-d6) δ 183.72, 152.25, 124.31, 120.48, 117.43, 115.51.

*(1,2,6,7-tetraoxo-1,2,6,7-tetrahydroindolo[7,6-g]indole-3,8-diyl)bis(ethane-2,1-diyl) bis(2-decyltetradecanoate) (**M1**)*: Bisisatin (1.00 g, 3.8 mmol) and potassium carbonate (1.95 g, 11.3 mmol) were dissolved in anhydrous DMF (60 mL) and stirred at 70 °C under nitrogen atmosphere for 1 h. To the resulting solution, 2-bromoethyl 2-decyltetradecanoate (5.36 g, 11.3 mmol) was added dropwise. The reaction mixture was heat at 70 °C with continuous stirring for 4 h. After completion, the reaction was cooled to room temperature and quenched

with saturated aqueous sodium bicarbonate. The aqueous layer was extracted with dichloromethane (DCM, 3 × 50 mL), and the combined organic layers were washed with water and brine, then dried over anhydrous magnesium sulfate. The solvent was removed under reduced pressure, and the crude product was purified by column chromatography (silica gel, DCM:hexane = 8:2, v/v), followed by precipitation in cold methanol. The product was collected by filtration to afford a dark blue solid (0.52 g, 13% yield). ^{1}H NMR (400 MHz, Chloroform-*d*) δ 8.25 (d, J = 8.7 Hz, 2H), 7.75 (d, J = 8.6 Hz, 2H), 4.48 (dt, J = 32.4, 6.3 Hz, 8H), 2.26 (m, 2H), 1.49 (m, 4H), 1.38 (m, 4H), 1.21 (m, 72H), 0.87 (t, J = 6.6 Hz, 12H). ^{13}C NMR (101 MHz, Chloroform-*d*) δ 181.96, 176.32, 159.34, 151.67, 127.11, 120.52, 120.05, 116.65, 60.84, 45.56, 42.23, 32.16, 31.92, 31.90, 29.68, 29.65, 29.60, 29.51, 29.46, 29.36, 29.33, 27.44, 22.69, 14.12. HRMS (m/z): (M·+H) calc. ($C_{66}H_{107}N_2O_8$): 1055.8022. found: 1055.8020. Raw ^{1}H NMR, ^{13}C NMR and MS data for M1 shown in Figure S3.

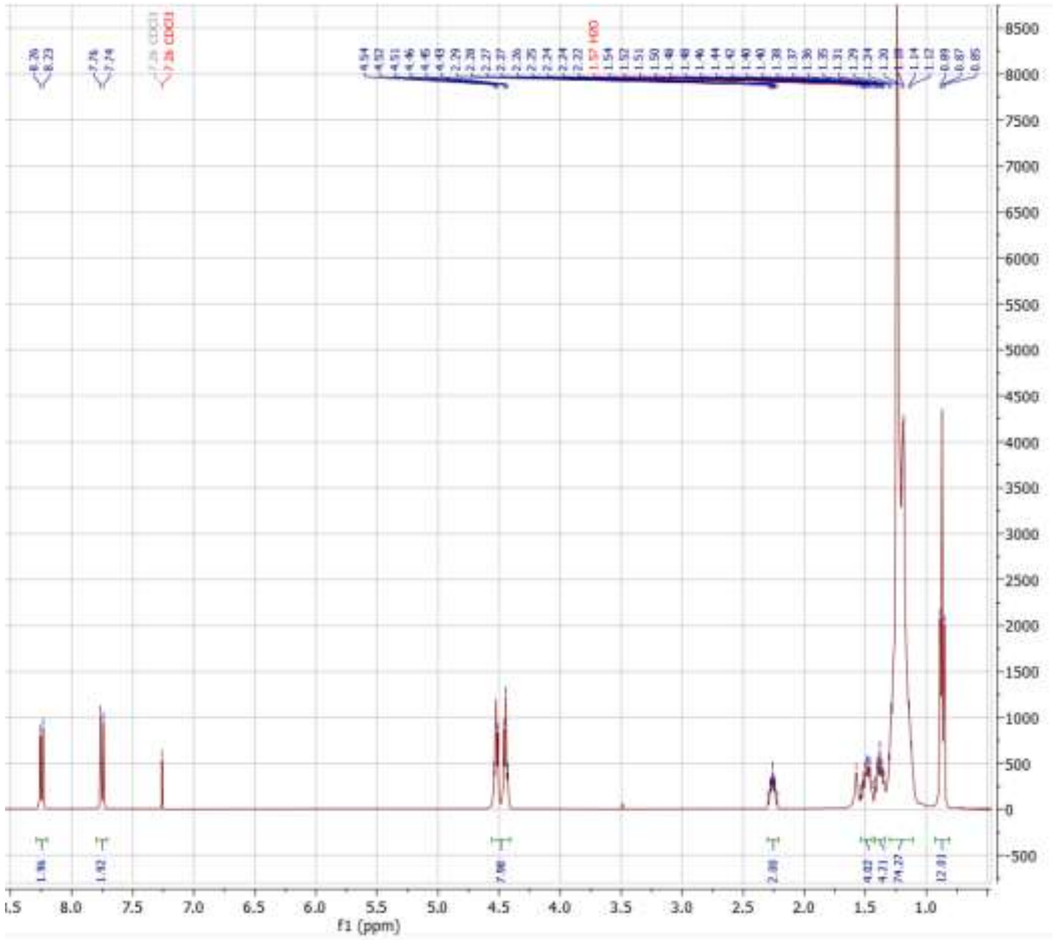

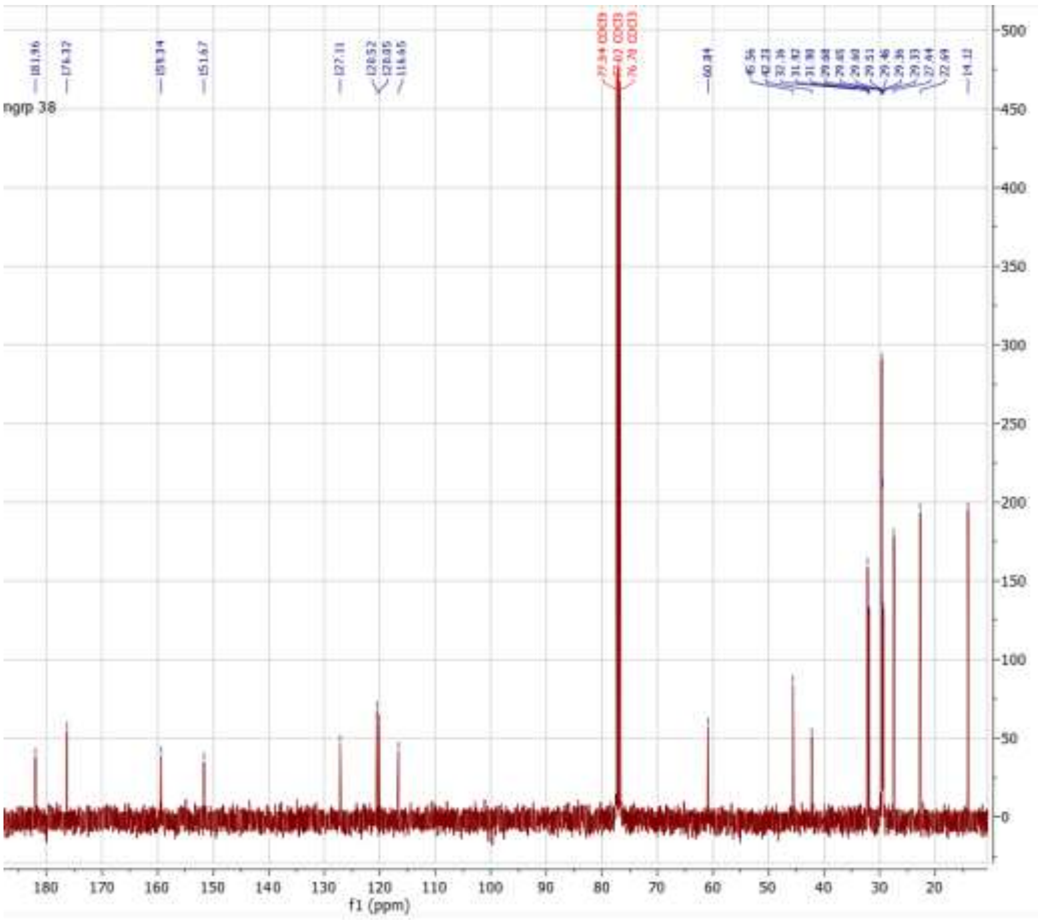

Proposed Formula	Adduct	Formula with Adduct	Calculated Mass (m/z)	Measured Mass (m/z)	Error (mmu)	Error (ppm)	Response
[illegible]	(+H)+	[illegible]	1055.8022	1055.8020	-0.20	-0.19	8172

The measured m/z value is consistent with your proposed formula for this sample.

Figure S3. Raw ^{1}H NMR, ^{13}C NMR and MS data of the monomer M1 for paNL.

3. Monomer M2 synthesis

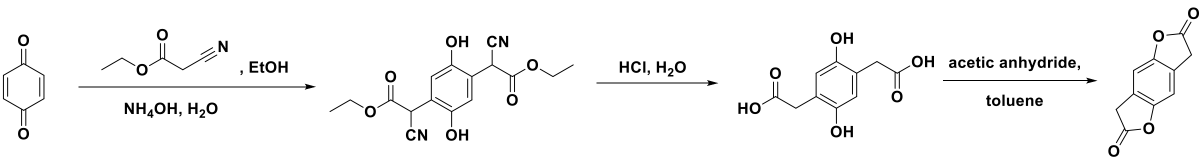

Figure S4. Synthesis of the monomer M2 for paNL.

Diethyl 2,2'-(2,5-dihydroxy-1,4-phenylene)bis(2-cyanoacetate) was synthesized following previously reported procedures[3]: Ethyl cyanoacetate (4.21 g, 4 ml, 37.25 mmol) in ethanol (15 ml) was added into a three-neck round-bottom flask equipped with two dropping funnels. Concentrated ammonium hydroxide (3.17 g, 3.5 ml, 90.62 mmol) was then added to the mixture.

In one dropping funnel, ammonium hydroxide (11.28 g, 12.5 ml, 322 mmol) in water (20 ml) was charged. A solution of ethyl cyanoacetate (6.4 g, 6 ml, 56 mmol) in ethanol (50 ml) was added p-benzoquinone (5 g, 46 mmol) in another flask and was stirred at 40 °C for half an hour before transferring to the other dropping funnel. The solutions in both dropping funnels were added to the flask at the same rate while stirring the reaction mixture. After completion of the addition, the resulting solution was stirred at room temperature for 1 hour. The precipitates were filtered and washed with ethanol and dried to yield diethyl 2,2'-(2,5-dihydroxy-1,4-phenylene)bis(2-cyanoacetate) (3.9 g, 26% yield) as dark purple solid.

2,2'-(2,5-dihydroxy-1,4-phenylene)diacetic acid was synthized following previously reported procedures[4]: Diethyl 2,2'-(2,5-dihydroxy-1,4-phenylene)bis(2-cyanoacetate) (3.9 g, 12 mmol)was dissolved in concentrated HCl (0.037 g, 25 ml, 1.02 mmol) and water (25 mL) in a RBF under nitrogen atmosphere. The reaction mixture was heat under reflux overnight. Upon completion, water (20 mL) and celite (2 g) were added to the hot solution and stirred for 5 minutes. The mixture was then rapidly filtered under suction while still hot. The clear filtrate was allowed to cool to room temperature, leading to the formation of white crystals (1.38 g, 52% yield).

3,7-dihydrobenzo[1,2-b:4,5-b']difuran-2,6-dione (M2) was synthized following previously reported procedures: To a residue of 2,2'-(2,5-dihydroxy-1,4-phenylene)diacetic acid (1.4 g, 6.2 mmol) in anhydrous toluene (75 mL), acetic anhydride (16.4 g, 15 mL, 195.96 mmol) was added under a nitrogen atmosphere. The reaction mixture was stirred at 100 °C overnight. After completion, the solvent was removed under reduced pressure. Chloroform was added to the resulting solid, and the mixture was filtered to remove any insoluble impurities. The filtrate was subsequently recrystallized from toluene to afford the final product as white crystals (0.1 g, 26% yield). ^{1}H NMR (400 MHz, DMSO -*d*) δ 7.24 (s, 1H), 3.95 (s, 2H). ^{13}C NMR (101 MHz, DMSO-*d*) δ 174.84, 150.58, 124.41, 107.81, 33.76.

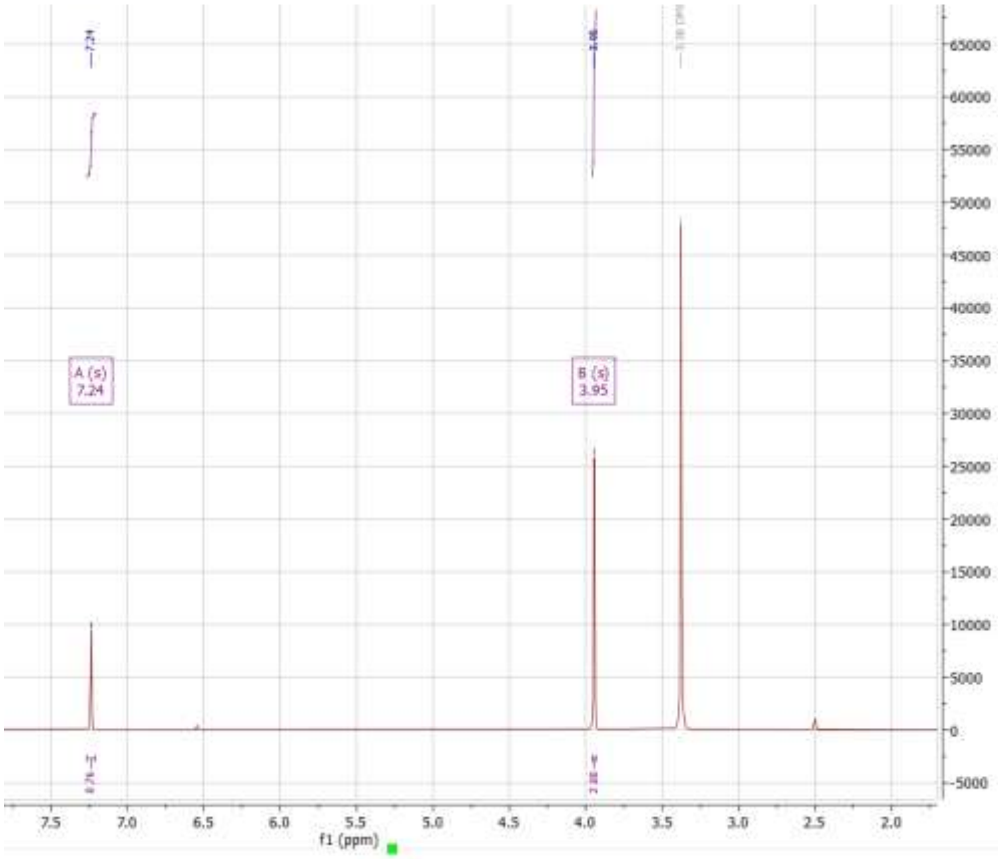
A (s)
7.24
B (s)
3.95
f1 (ppm)

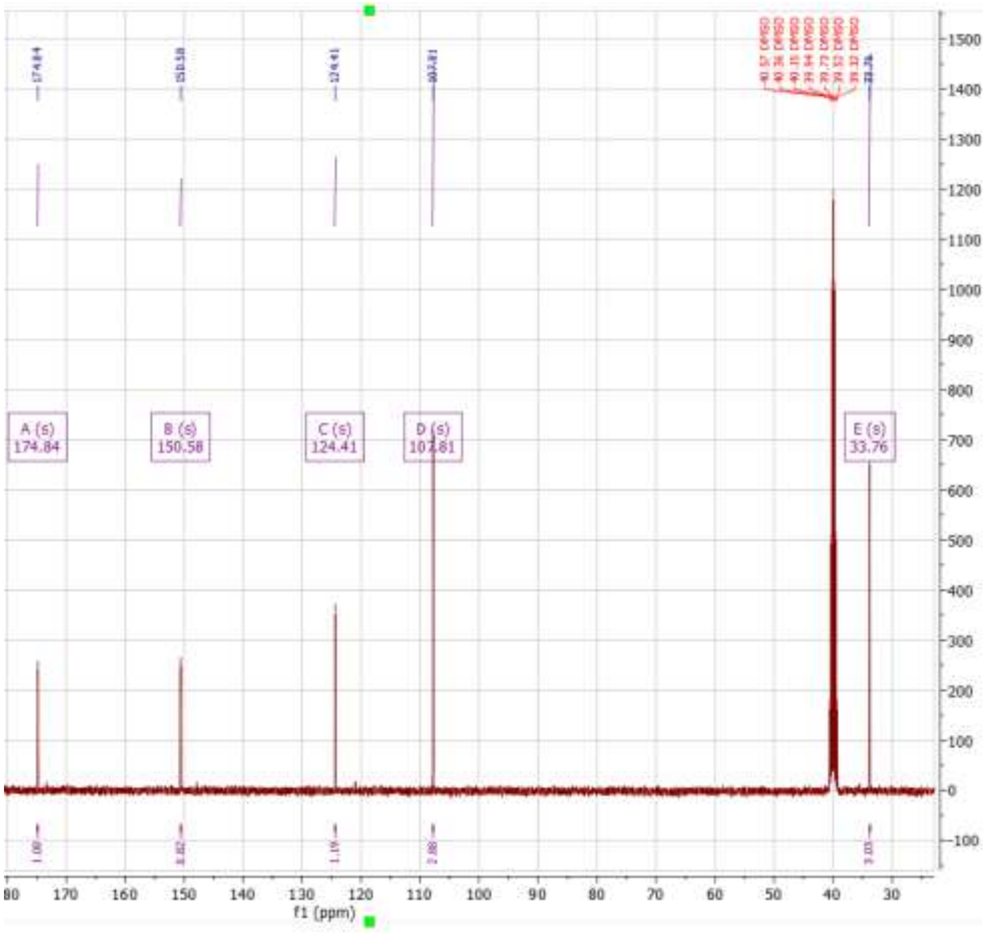
A (s)
174.84
B (s)
150.58
C (s)
124.41
D (s)
107.81
E (s)
33.76
f1 (ppm)

Figure S5. Raw ^{1}H NMR and ^{13}C NMR of the monomer M2 for paNL.

4. Polymer paNL synthesis

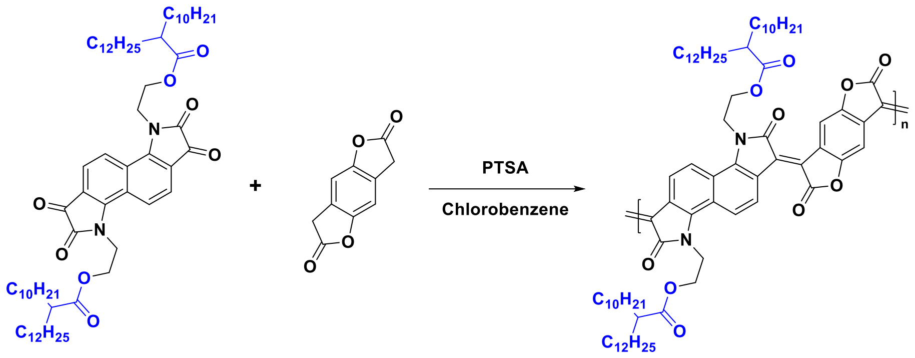

paNL: A microwave vial was charged with M1 (117.24 mg, 0.111 mmol), 3,7-dihydrobenzo[1,2-b:4,5-b']difuran-2,6-dione (21.12 mg, 0.111 mmol) and p-toluene sulfonic acid monohydrate (12 mg, 0.063 mmol). Anhydrous toluene (3 mL) was injected. The resulting solution was purged with nitrogen for 30 min and the reaction was heated to 130 °C for 1 day. The reaction mixture changed colour from dark purple to dark blue over the polymerization period. The crude polymer was precipitated in methanol and purified by Soxhlet extraction with methanol, ethyl acetate, hexane and chloroform. The chloroform fraction was collected and the solvent was removed under vacuum. Afterwards, the polymer precipitated into methanol, filtered and dried. Yield: 113 mg, 85 %, dark blue solid. GPC (Chlorobenzene, 80 °C): Mn 15.8 kDa, PDI 2.06. ^{1}H NMR (400 MHz, Chloroform-*d*) δ 8.29 - 9.15 ppm (d), 3.62 – 5.17 ppm (m), 2.54 - 3.02 ppm (m), 0.46-2.32 ppm (broad).

Figure S6. Synthesis of the polymer paNL.

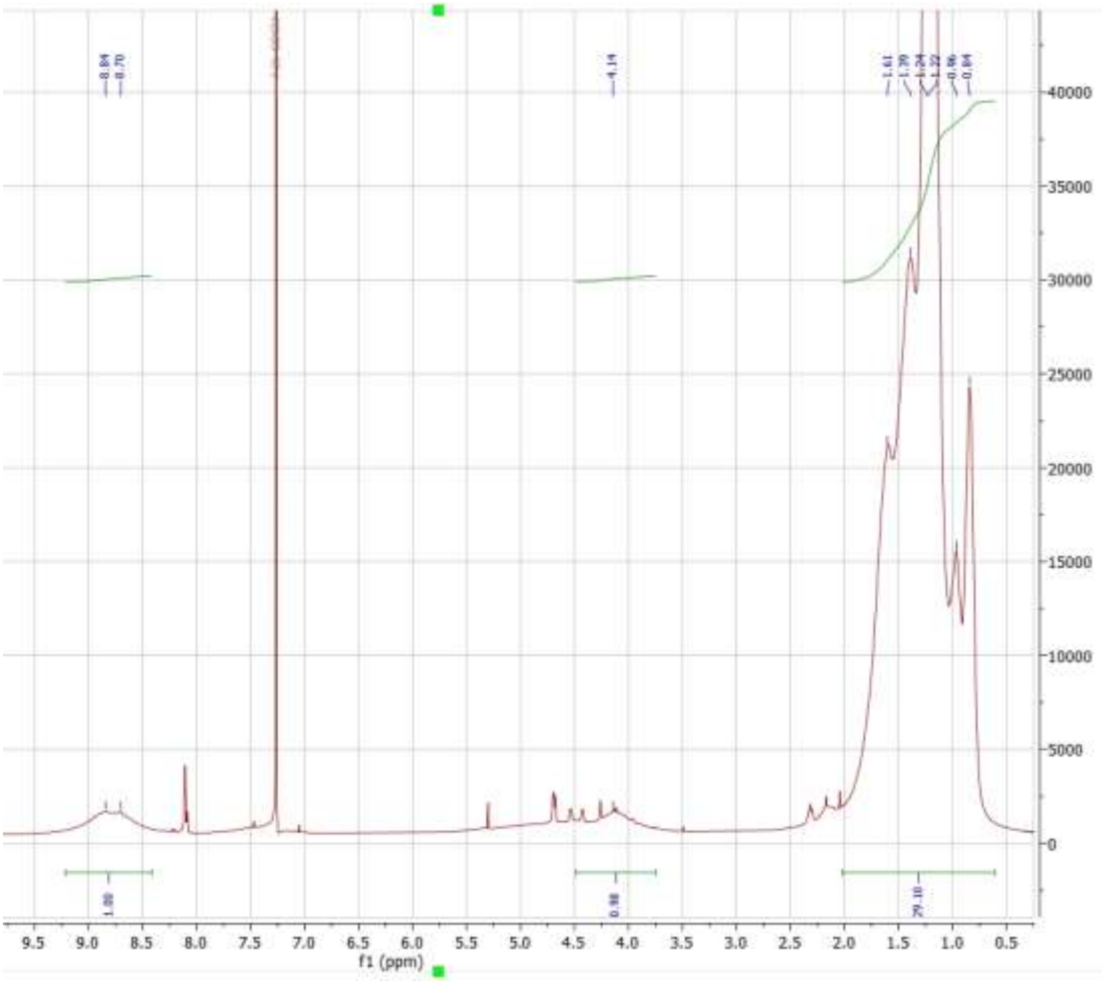

Figure S7. Raw ^{1}H NMR of paNL.

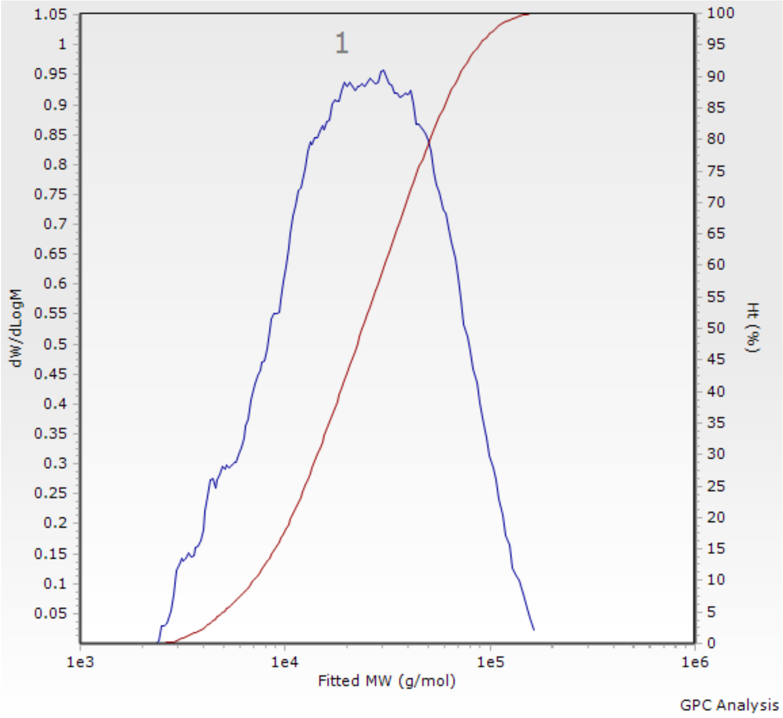

Figure S8. Raw GPC trace of paNL.

C. Annealing procedure investigations

Thermogravimetric analysis (TGA) measurements were conducted using a TA Instruments TGA5500-0611 under a nitrogen atmosphere with a flow rate of 10 mL/min. Samples were initially equilibrated at 40.00 °C, followed by a heating ramp of 10.00 °C/min to 300.00 °C. The temperature was then held isothermally at 300.00 °C for 6 hours. Subsequently, the sample was heated at a rate of 10.00 °C/min to 350.00 °C, followed by cooling to 40.00 °C at a rate of 10.00 °C/min.

Condition screenings for post-deposition thermal treatments were conducted using thermogravimetric analysis (TGA). Initially, the paNL powder was heated to 800°C. However, a continuous mass loss was observed from 250 °C to 800 °C, likely due to the high density of the cleaved side chains, which prolonged their evaporation. (Figure S9)

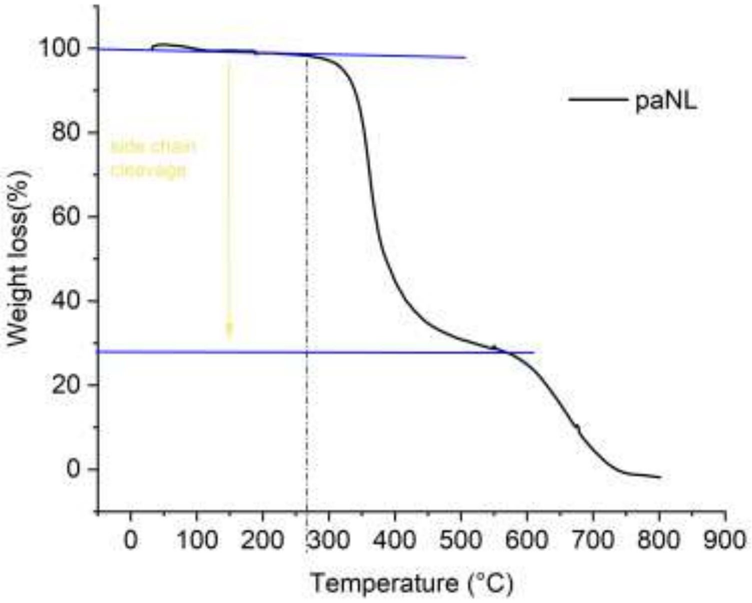

Figure S9. TGA for paNL from 25 to 600 °C

To address this, the heating process was divided into two steps: i) The powder was heat to the expected cleavage temperature at a rate of 10 °C/min. ii) It was then hold at the cleavage temperature for a long time (12 hours) to check if the mass dropped into the expected value. Different temperatures have been carried out in 250 °C (Figure S10), 270 °C (Figure S11) and 300 °C (Figure S12), respectively. In both 250 °C and 270 °C, paNL started to decompose. However, the rate of decrease is too slow that after heating at 250 °C or 270 °C for 12 hours,

the weight percentage only dropped to 71% and 58%, respectively. Both results illustrate that both temperatures are not enough to fully cleave the side chains.

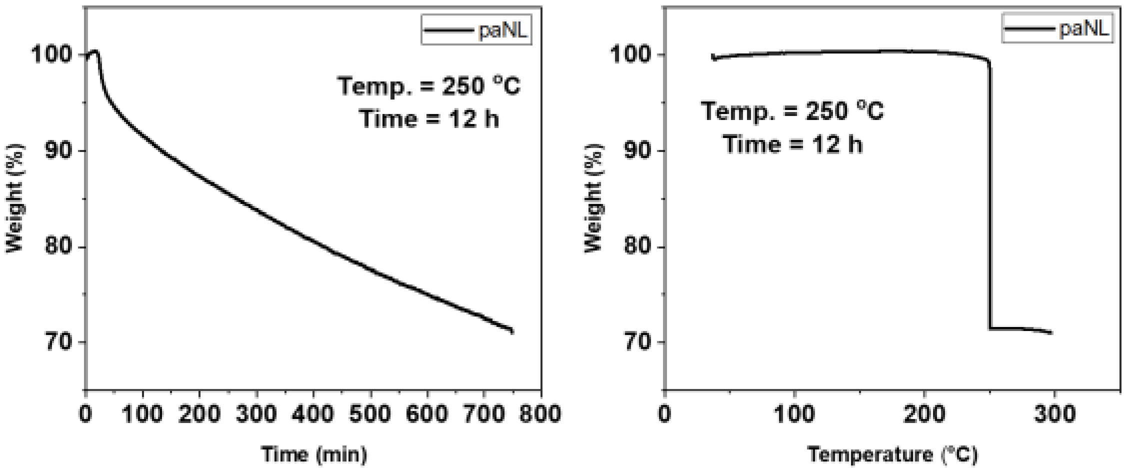

Figure S10. TGA for paNL holding at 250 °C

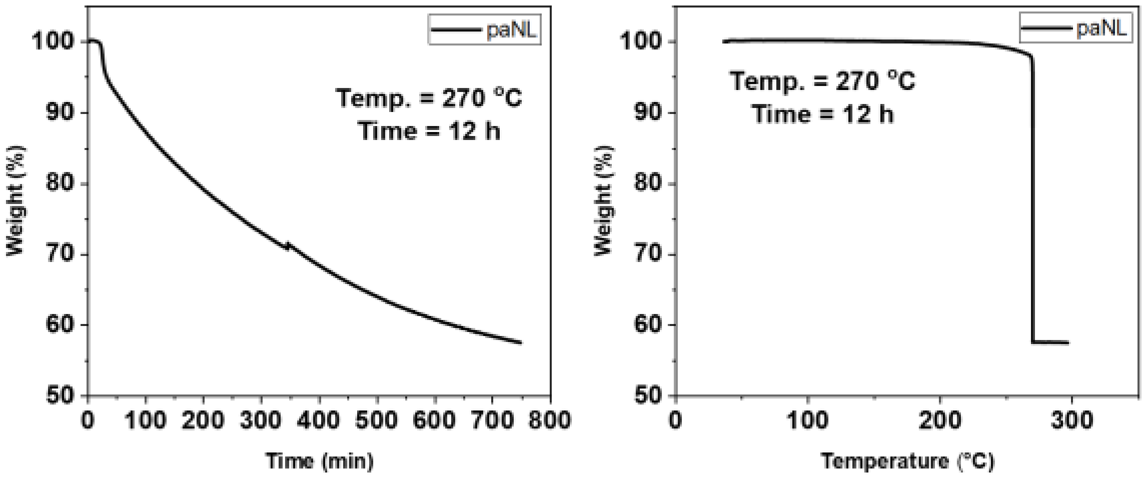

Figure S11. TGA for paNL holding at 270 °C

From the TGA result for paNL holding at 300**°C**, it is clearly shown that approximately 60% mass loss was observed in step 2, which is consistent with the mass of the side chains of the polymer, and hence this temperature was selected for the post deposition cleavage. Furthermore, no additional mass loss was observed between 300°C and 350°C, indicating that the cleaved polymer possesses good thermal stability.

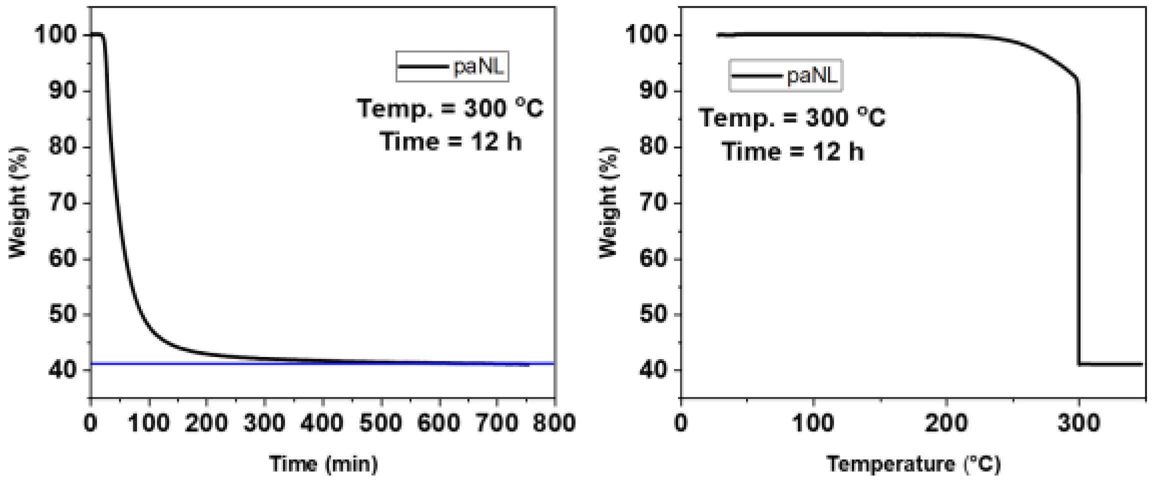

Figure S12. TGA for paNL holding at 300°C

D. Preparation and investigation of peNL

1. Polymer peNL synthesis

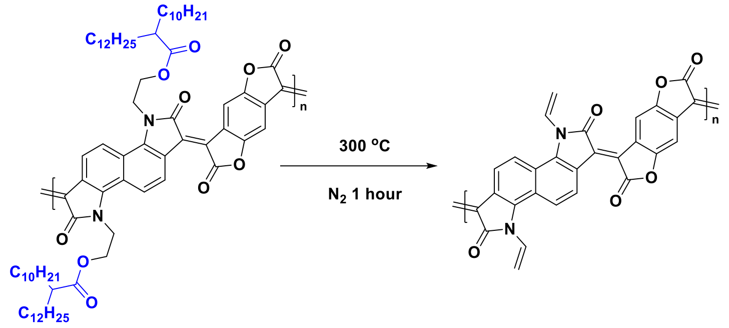

Figure S13. Synthesis of the polymer paNL**.**

The synthetic procedure for thin film peNL: The powder of paNL were dissolved in $CHCl_3$ with concentration of 10 mg/mL and stirred overnight to ensure complete dissolution**.** The polymer solution was then spin-coated at 1000 rpm for 60 seconds in ambient air on a substrate. The paNL film was then transferred into a glovebox under the protection of nitrogen and heat at 300**°C** for one hour to form peNL film.

2. Eliminated side product characterisation

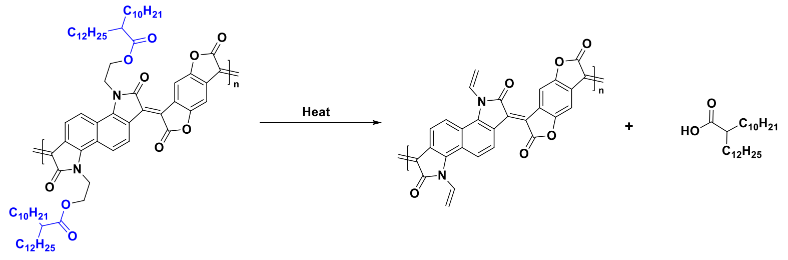

Figure S14. Elimination of paNL to yield 2-decyltetradecanoic acid.

2-decyltetradecanoic acid: The eliminated alkyl carboxylic acid side product was synthesized by heating paNL. The eliminated side chain was collected by condensing during the thermal treatment process. Its chemical structure determined by ^{1}H NMR (400 MHz, CDCl3) δ 2.34 (m, 1H), 1.59 (m, 2H), 1.44 (m, 2H), 1.25 (m, 36H), 0.88 (t, J = 6.6 Hz, 6H) and HRMS (m/z): ($M^{\cdot+}$ H) calc. ($C_{68}H_{114}N_2O_4$): 369.3727. found: 369.3709.

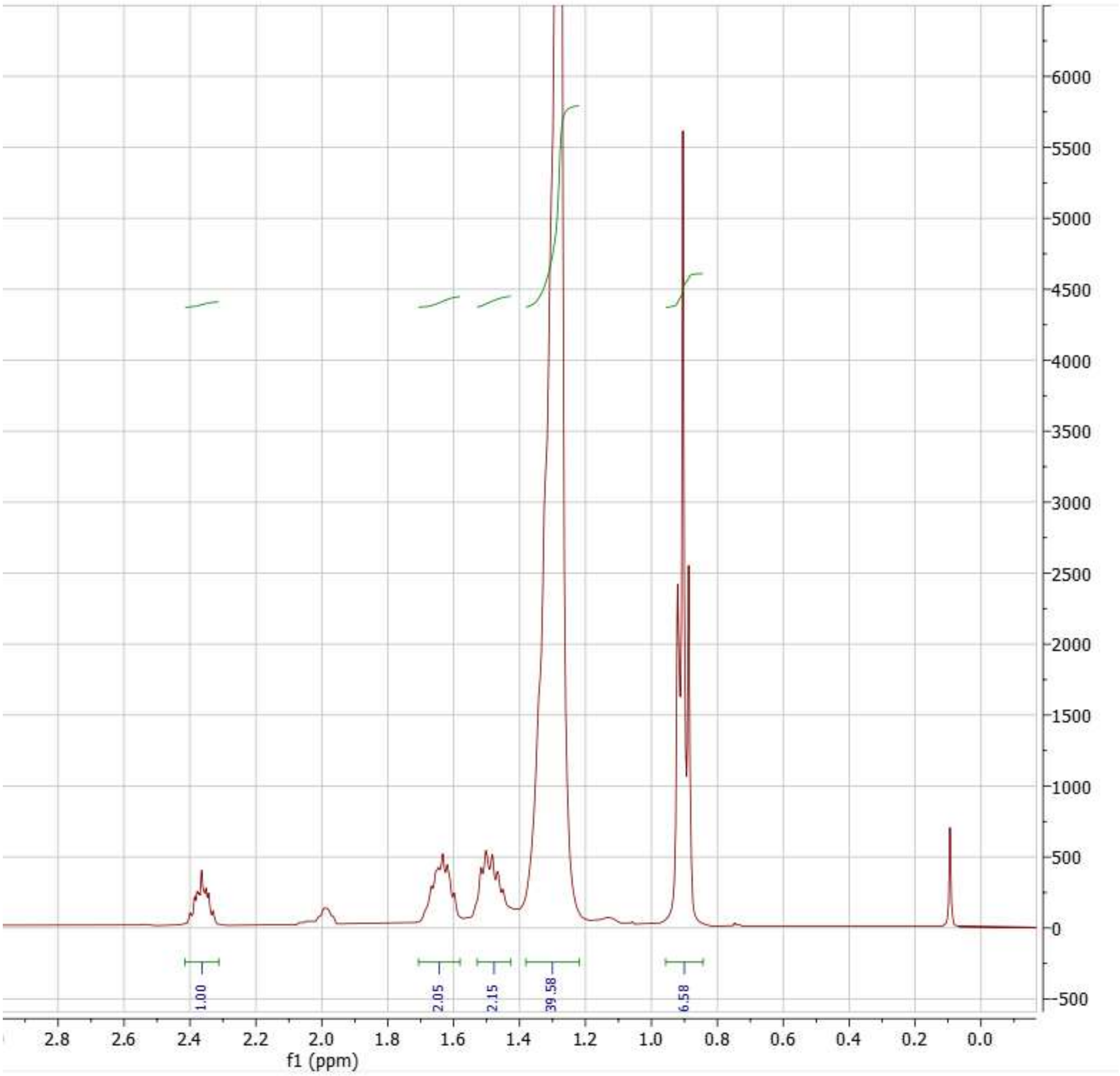

Measured Mass	Calculated Mass	Error (mDa)	Error (ppm)	Formula $[M+H]^+$	Response
369.3709	369.3727	-1.81	-4.90	$C_{24}H_{49}O_2$	28711

Figure S15. ^{1}H NMR and HRMS of the byproducts including 2-decyltetradecanoic acid cleaved from thermal treatment of paNL.

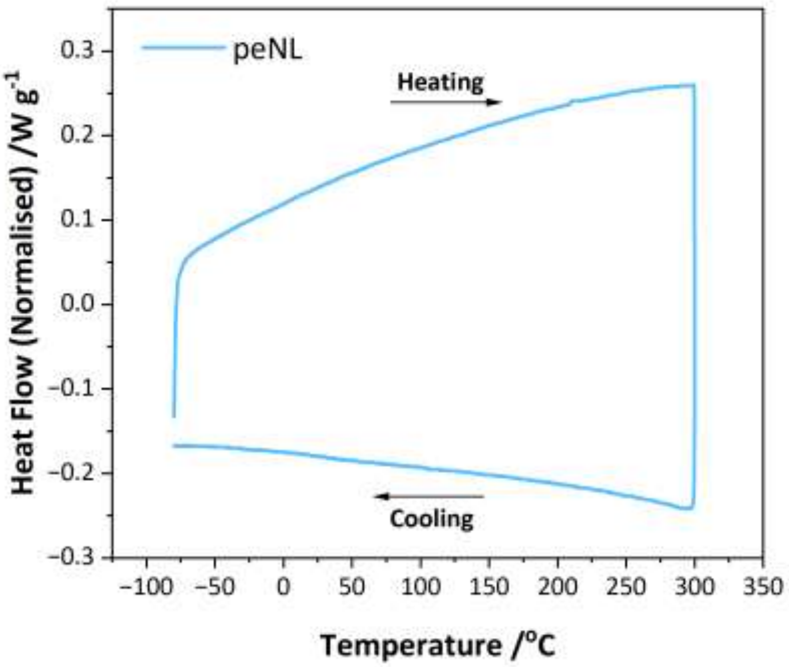

Figure S16. DSC heating and cooling circles of peNL.

E. Film annealing procedure: X-ray photoelectron spectroscopy (XPS) analysis

X-ray Photoelectron Spectroscopy (XPS) measurements were performed using a Scienta-200 hemispherical analyzer with a monochromatized Al Kα source (photon energy E_{ph} = 1486.6 eV). Spectra were calibrated by referencing the binding energy scale to the Fermi level and the Au $4f_{\frac{7}{2}}$ peak position of an Ar^{+} ion sputter-cleaned gold foil. The analysis was conducted under ultra-high vacuum conditions, with a base pressure below 1×10^{-9} mbar.

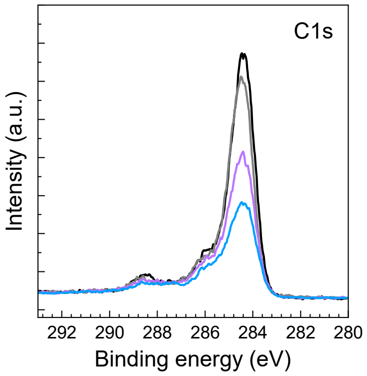

Figure S17. XPS C 1s and O 1s spectra of paNL and thermal treatments at 260 °C for 1 hour, 300 °C for 30 minutes, and 300 °C for 1 hour, normalized to the constant N 1s peak area for accurate comparison. Peak intensities were corrected based on atomic sensitivity factors.

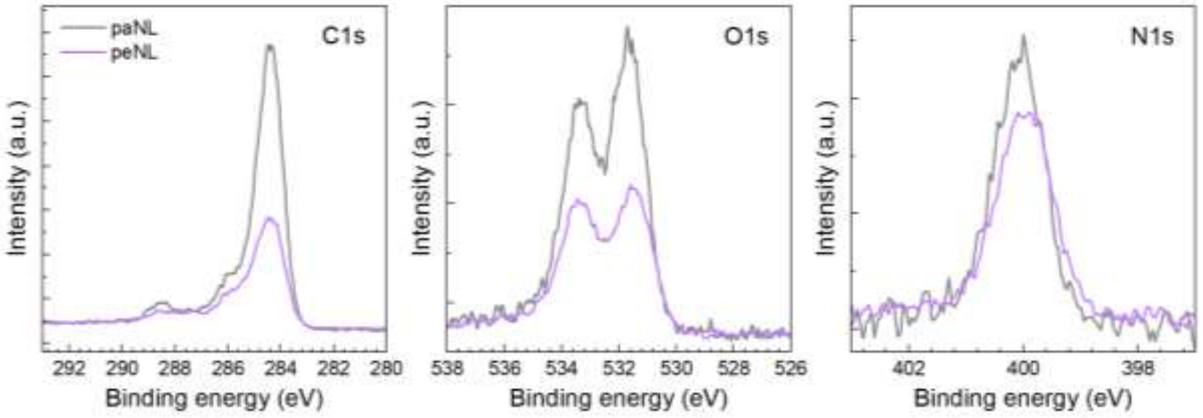

Figure S18. XPS C 1s and O 1s spectra of paNL and peNL, normalized to the constant N 1s peak area for accurate comparison. Peak intensities were corrected based on atomic sensitivity factors.

Table S1. XPS peak fitting parameters of C 1s, O 1s, and N 1s spectra of paNL and peNL.

	Peak position					Peak FWHM					Peak area percentage				
C1s	C-C, C=C	C-N	C-O	N-C=O	O-C=O	C-C, C=C	C-N	C-O	N-C=O	O-C=O	C-C, C=C	C-N	C-O	N-C=O	O-C=O
paNL	284.41	285.54	285.98	287	288.48	1.09	1.09	1.09	1.09	1.09	0.755	0.070	0.070	0.035	0.070
peNL	284.4	285.63	286.23	287.49	288.63	1.21	1.21	1.21	1.21	1.21	0.612	0.155	0.078	0.078	0.078
N1s	C-N-C														
paNL	400.08					1.03									
peNL	399.96					1.13									
O1s	C=O···H	C-O-C	O-C=O	N-C=O		C=O···H	C-O-C	O-C=O	N-C=O		C=O···H	C-O-C	O-C=O	N-C=O	
paNL		533.42	531.91	531.42			1.23	1.23	1.23			0.4	0.4	0.2	
peNL	534.13	533.3	531.73	531.25		1.58	1.33	1.33	1.33		0.111	0.337	0.337	0.216	

F. Thin film ionisation potential and electron affinity characterisations

The normalized thin film UV-Vis-near-infrared (NIR) absorption spectra and Photoelectron spectrometer in air (PESA) were measured in thin films of paNL and peNL.

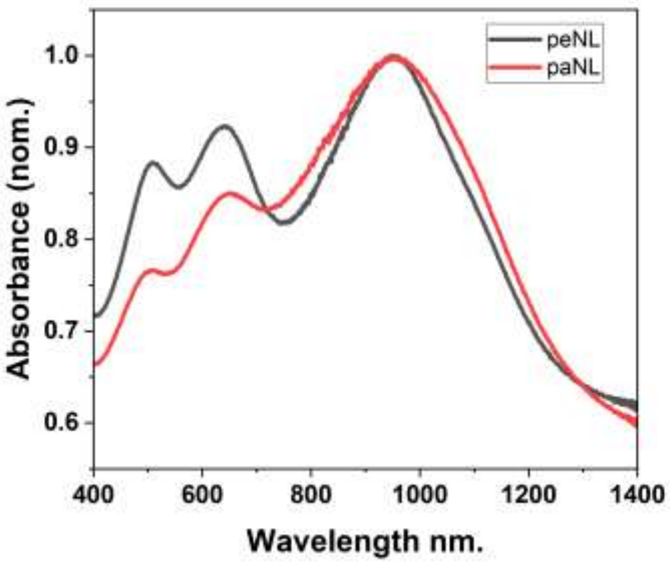

Figure S19. Normalized UV-Vis-near-infrared (NIR) absorption spectra for paNL and peNL thin films.

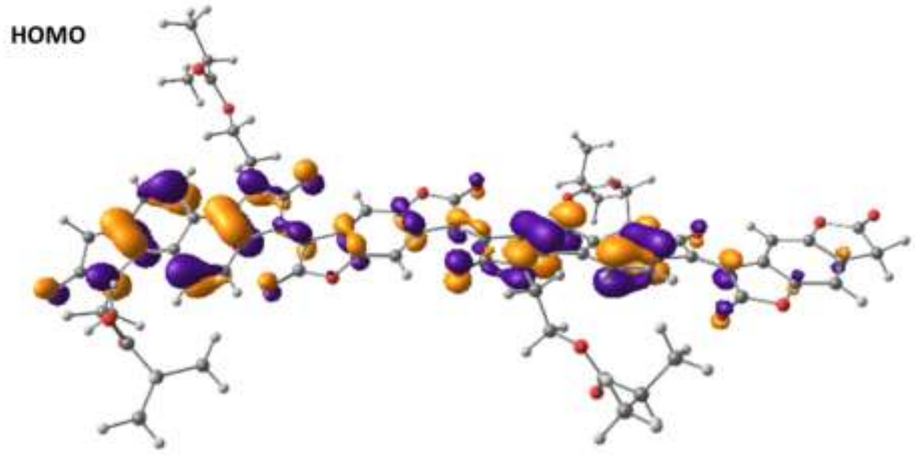

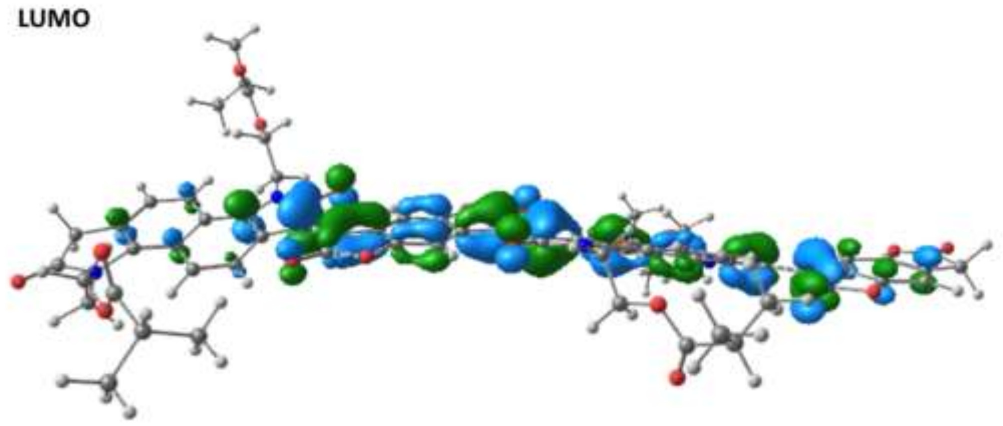

Figure S20. Density Functional Theory calculation (DFT) for paNL.

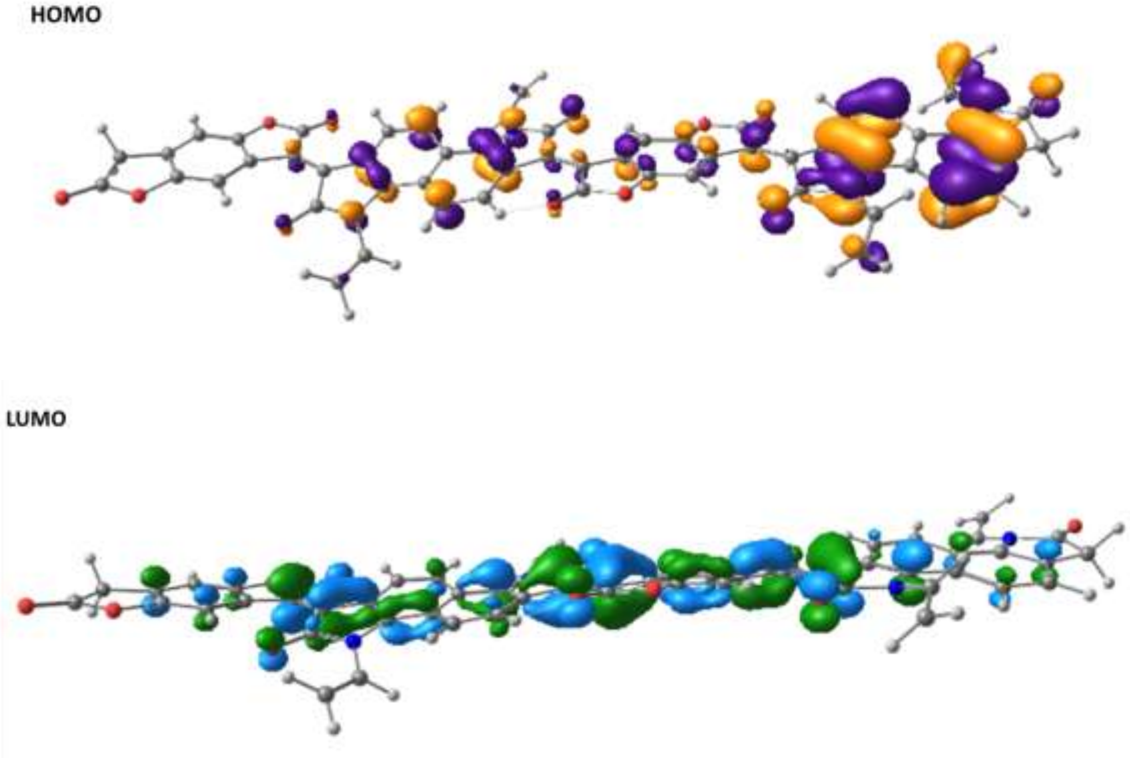

Figure S21. Density Functional Theory calculation (DFT) for peNL.

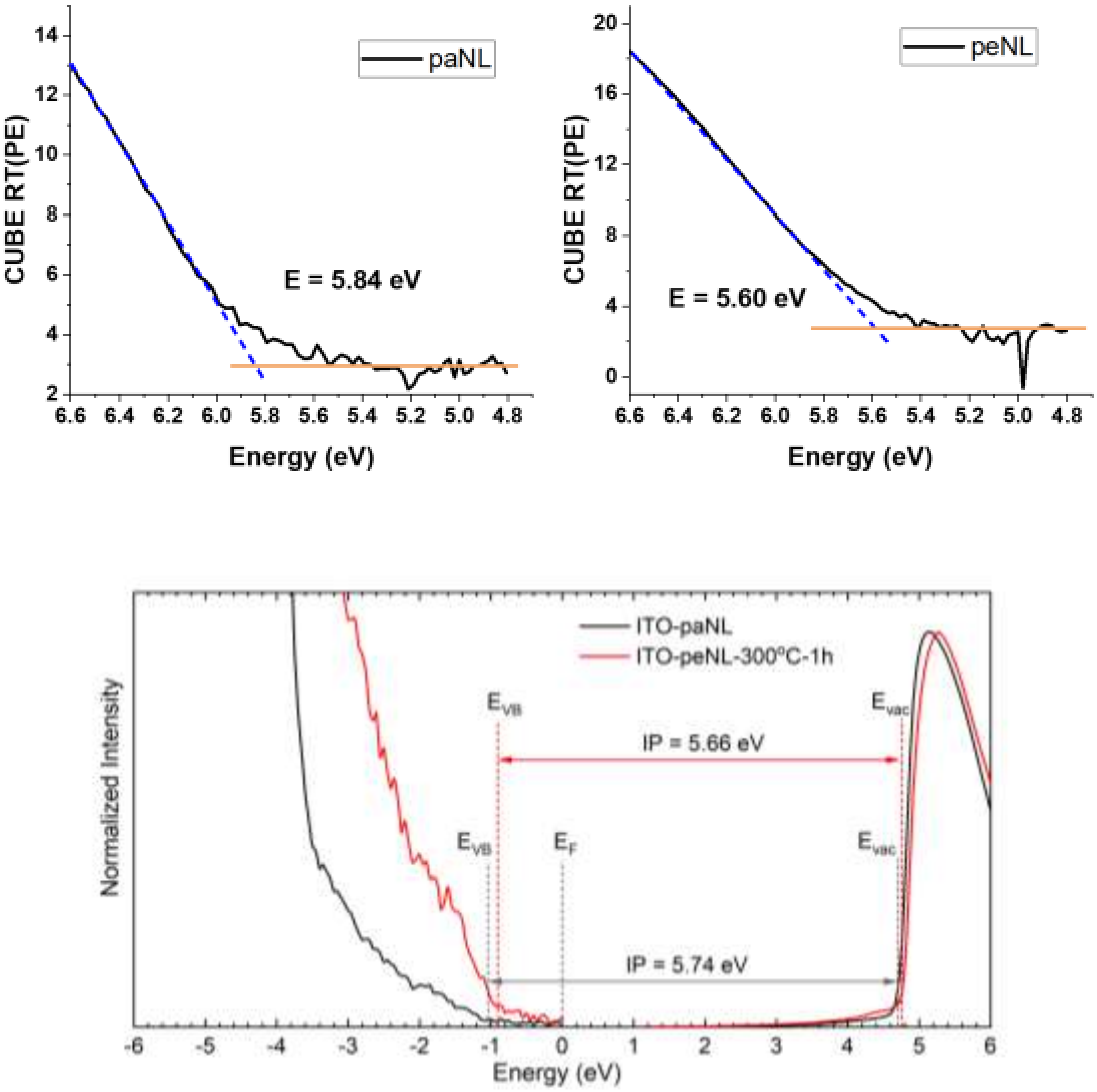

Figure S22. PESA and UPS of paNL and peNL thin films.

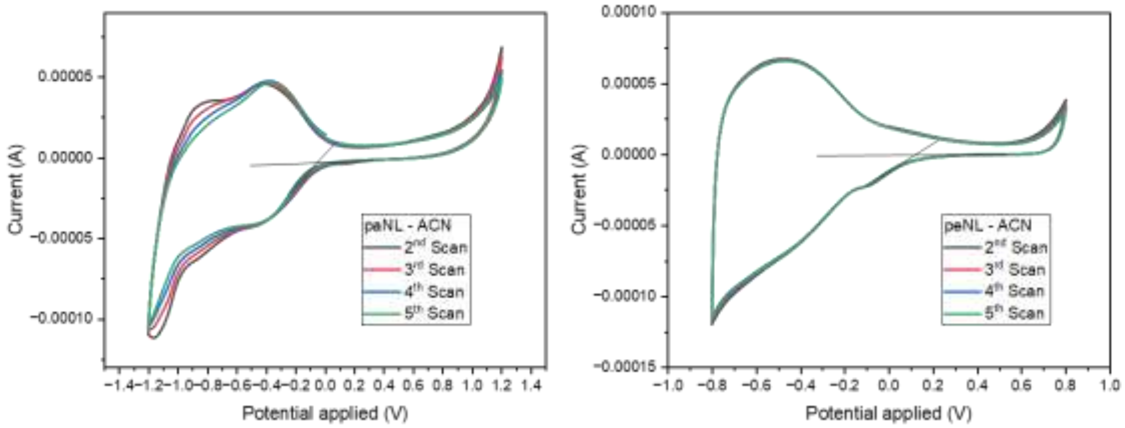

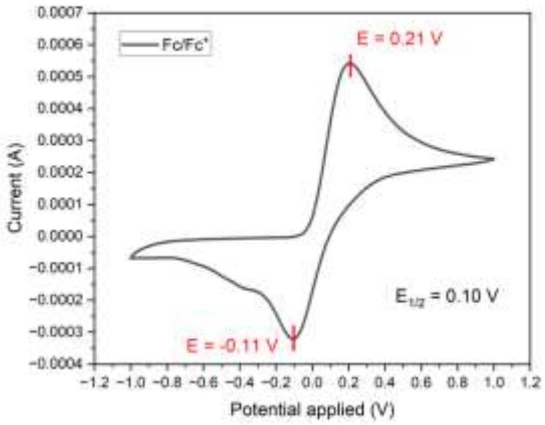

Figure S23. Cyclic voltammetry (CV) of paNL and peNL thin films and Ferrocene in ACN.

Table S2. Optical summary of paNL and peNL:

Polymer	$\lambda_{max,film}$	E_{opt} (eV)[a]	IP (eV)[b]	IP (eV)[c]	$E_{re,org}$ (eV)	EA (eV)[d]	$E_{onset,aq}$ (eV)
paNL	949	1.01	5.8	5.74	-0.05	4.65	-0.3
peNL	945	0.99	5.6	5.66	0.11	4.8	-0.1

[a] Determined from the optical absorption onset. [b] Determined from PESA. [c] Determined from UPS. [d] Calculated from EA (eV) = (4.8 - E_{FC} + $E_{re,org}$) where E_{FC} is the half-way potential of the ferrocene/ferrocenium (Fc/Fc^+) redox couple. The electron affinities (EAs) were calculated by subtracting the optical band gap from the IP values.

G. Spectroelectrochemistry (SEC) spectra

In situ Spectroelectrochemistry (SEC) measurements were conducted in an airtight glass three-electrode cell. Neat films coated on ITO substrates were employed as the working electrode, with Ag/AgCl wire as the quasi-reference electrode and a platinum wire as the counter electrode. The electrolyte consisted of 0.1 M NaCl electrolyte. A collimated 10 mW halogen lamp (Ocean) served as the light source. The light re-collimated and directed into an optical fiber for detection. A near-infrared CCD camera was used to capture spectra in the near-infrared range (1000 nm to 1700 nm) using an Ocean Insight NIR Quest. Potentials were controlled using an IVIUM VERTEX potentiostat and custom-built LABVIEW software.

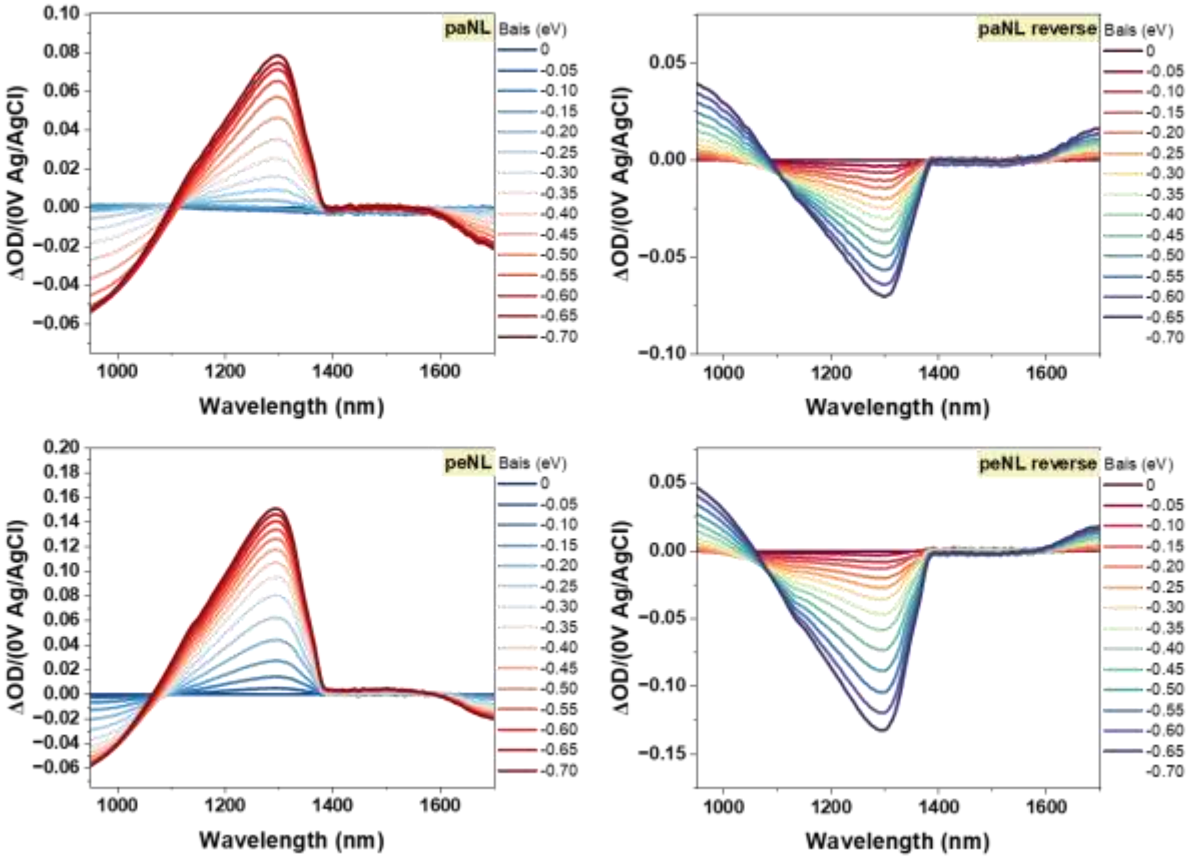

Figure S24. Spectroelectrochemistry: Potential dependent UV/Vis-NIR absorption spectra and reverse spectra of paNL and peNL.

H. Supporting Figures for hydrophilicity and swelling behaviour of paNL and peNL

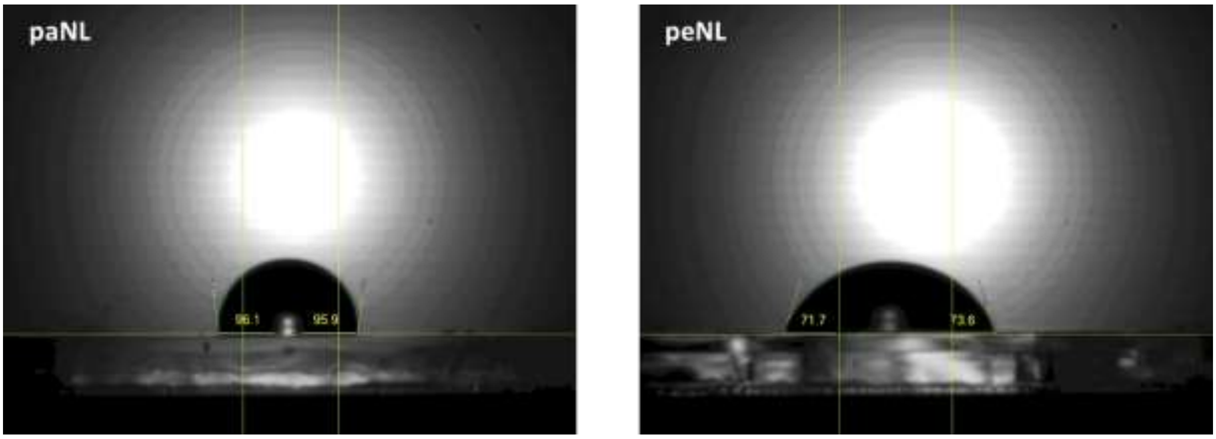

Figure S25. Contact angle measurement of paNL and peNL.

Table S3. Contact angle measurement summary of paNL and peNL.

	paNL	peNL
Test 1	96.3	71.6
Test 2	97.5	73.1
Test 3	95.1	74.9

Atomic force microscopy (AFM): Tapping mode AFM was performed in both air and fluid using a Bruker Multimode 8. Silicon probes (resonant frequency 75 kHz, spring constant 2.8 N/m) were used for measurements in air. Silicon nitride probes (resonant frequency 150 kHz, spring constant 0.7 N/m) were used for measurements in fluid. AFM fluid operation was performed using the same film samples measured in the dry state. The samples were placed in a tapping mode fluid cell, and a 0.1 M NaCl electrolyte solution was introduced to fully immerse the films under aqueous conditions.

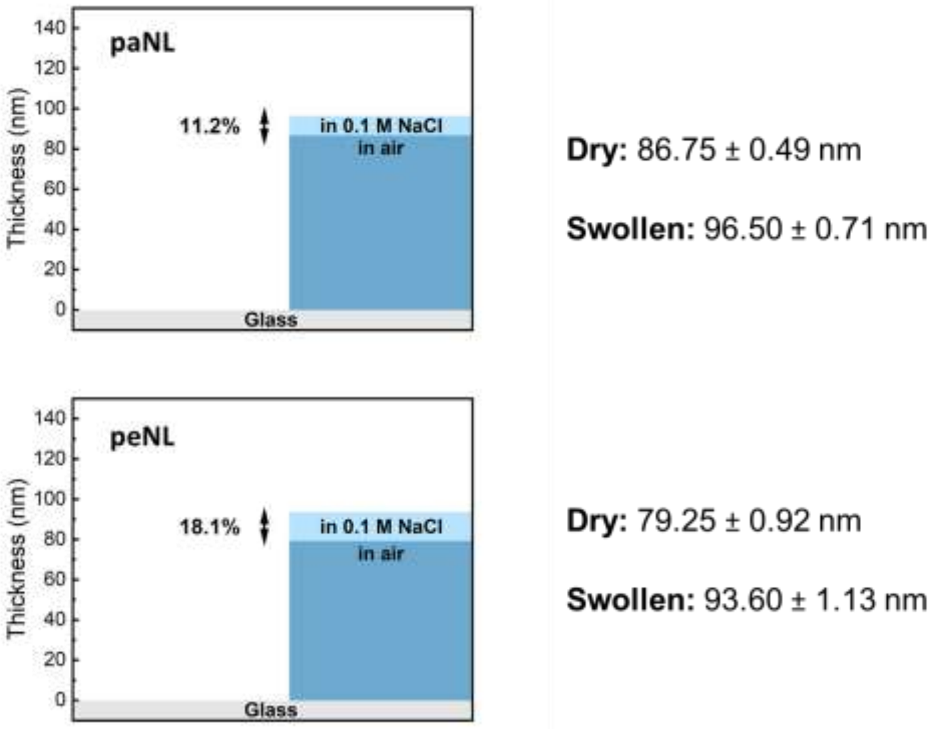

Figure S26. Film Swelling Behavior via Liquid AFM for paNL and peNL.

I. Supporting Figures for OECT

OECT Device Characterization: For both transient and steady state OECT characterization a dual channel Keithley source meter controlled by a custom-made Labview code. Measurements performed using an Ag/AgCl pellet (D = 1 mm × H = 3 mm – World Precision instrument EP1) as the gate electrode. The data collected was used to plot the output characteristics along with the transfer characteristics to extract the transconductance curve, which was in turn used for the extraction of the mobility (μ) using the Bernard-Malliaras[5] for the saturation region of operation,

$$g_m = \frac{Wd\mu C^*}{L}(V_G - V_{TH})$$

where g_m is the transconductance, W and L is the width and the length of the interdigitated contacts respectively, d is the is the thickness of the polymer film, μ is the mobility, C^* is the volumetric capacitance, V_G is the applied gate voltage and V_{TH} is the threshold voltage. The slope of linear portion of g_m vs V_G curve was used to determine the μC^* product followed by the computation of mobility using the previously obtained C^* values from the EIS characterisation.

For the calculation of the response time, the transient response of the OECT was obtained by obtaining drain current (I_D) values under a constant drain voltage (V_D) of 0.6 V and a gate voltage (V_G) pulse train with the amplitude of 0.4 V and time-period of 6 seconds. The I_D response at the sixth gate pulse was used to onto an exponential decay function to extract the time constant (τ) which is the measure of the response time of the device.

OECT Substrate Fabrication: OECTs were fabricated with the interdigitated electrode architecture as previously described, with the detailed layout illustrated in Figure S30.[12] The interdigitated electrodes were fabricated via a liftoff process with the bilayer resists (SF11 Kayaku Advanced Materials; AZP4110, Integrated Micro Materials). This was followed by UV exposure using a Suss MA6 Mask Aligner with subsequent development in AZ400K developer (Integrated Micro Materials) for AZP4110 and 101B developer (Kayaku Advanced Materials) for SF11. Ti/Au were deposited by thermal evaporation, followed by liftoff in an N-methyl-2-pyrrolidone (NMP) bath at 60 °C. The polymer paNL solution (10 mg/mL in chloroform) was spin-coated onto the interdigitated substrates under dynamic conditions at 1000 rpm with an acceleration of 500 rpm for 1 minute.

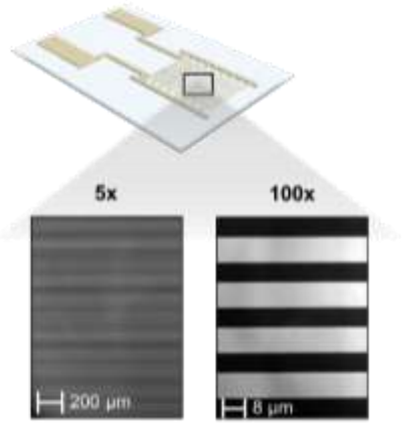

Figure S27. OECT IDE Architecture.

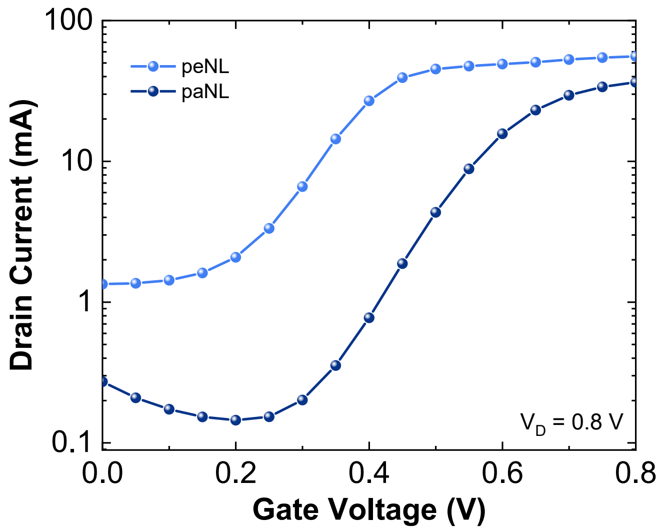

Figure S28. Logarithmic-scale transfer characteristics for paNL and peNL.

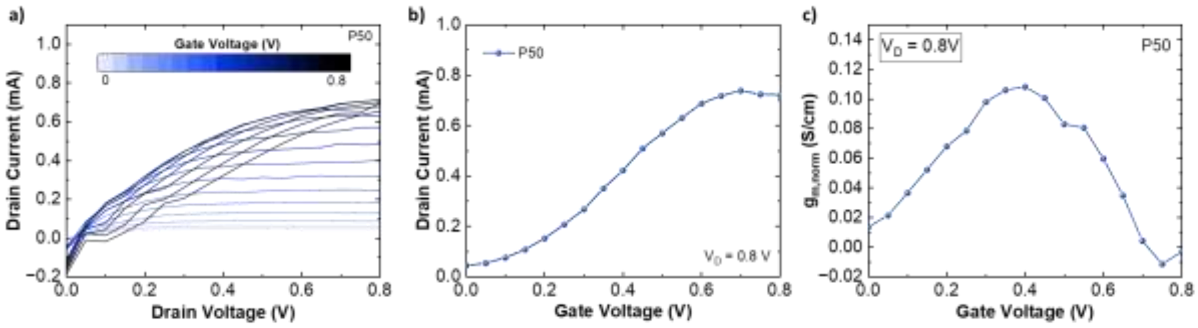

Figure S29. OECT performance of an analogous glycolated polymer P50 on the IDE substrate. a) Representative output characteristic curves and b) standard transfer curve of P50. c) Representative geometry normalized transconductance vs gate voltage curve for P50 using

the dimensions of OECTs – i.e. W/L = 3750. The thicknesses of the channels was measured at 39 nm for P50.

Table S4. Polymer OECT performance of analogous glycolatyed polymer P50 on literature substrate[8] and IDE substrate.

P50	g_m [µS] a)	g_m / (Wd/L) [S cm^{-1}] b)
Literature	730	0.35 ± 0.05
IDE substrate	1580	0.11

a Peak transconductance extracted from the transfer curve at saturation regime. b Extracted from the slope of OECT transfer curves and normalized by channel thickness and aspect ratio.

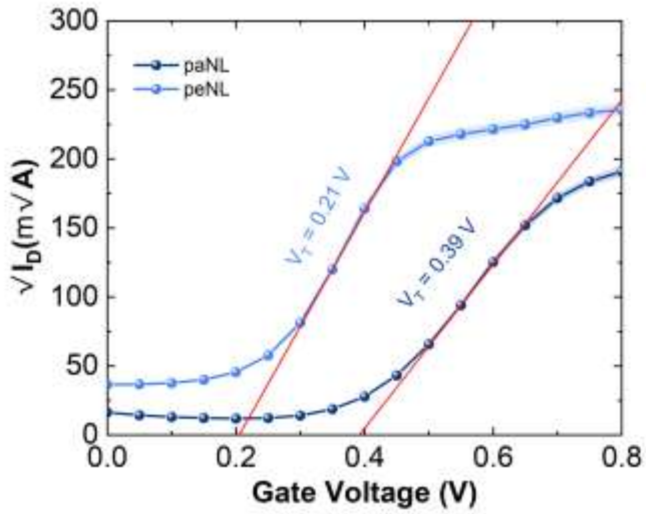

Figure S30. √ID versus V_G curves for the polymer OECT channel for peNL (blue lines) and paNL (black lines). The threshold voltage is found to be 0.21V and 0.39 respectively.

Table S5. Comparison of reported n-type mixed OECT figure of merits of state-of-the-art OMIEC materials

OMIEC	$\frac{g_m}{Wd/L}$ [S cm^{-1}]	µC* [Fcm^{-1} V^{-1}s^{-1}]	C* [Fcm^{-3}]	µ [$\frac{cm^2}{V\,s}$]	V_T [V]	Ref.
PgNaN	0.212	0.652±0.107	100±6	(6.50±1.01) × 10^{-3}	-	[6]

PgNgN	0.007	0.046±0.024	239±97	$(1.89\pm0.23) \times 10^{-4}$	-	[6]
p(g7NC10N)	0.37	1.83	153	0.012	0.3	[7]
P-75	5.5	14.76	98.4	0.15	-0.02	[8]
P-90	0.009	0.0343	261.5	0.000074	0.24	[9]
BBL	0.359	0.65	930	0.0007	0.21	[10]
BBL152	1.11	2.59	589	0.0044	0.15	[11]
hPDI[3]	0.35	1	-	-	-	[12]
3gY	-	2.7	155	0.018	0.4	[13]
f-BTI2g-TVTCN	12.8	41.3	170	0.24	0.68	[14]
PBFDO	-	180	-	-	-	[15]
P(gTDPP2FT)	0.35	54.8	156	0.35	0.64	[16]
f-BTI2g-TVTF	22.6	90.2	222	0.41	0.75	[17]
Pg5Tz-5-DPP	31.9	96.3	243	0.38	0.31	[18]
n-PT3	40.4	91.8	103	0.89	0.56	[19]
gDPP-tB0.1	26.9	118	203	0.59	0.77	[20]
p(C2F-V)	25.6	107.5	118	0.91	0.02	[21]
f-BseI2g-SVSCN	71.4	191.2	387	0.36	0.68	[22]
**paNL**	**12.69**	**46.87**	**115**	**0.407**	**0.39**	**This Work**
**peNL**	**28.38**	**136.23**	**227**	**0.6**	**0.21**	

Electrochemical Characterization: Electrochemical Impedance Spectroscopy versus frequency measurements were performed with the PalmSens4 potentiostat (PalmSense BV.) using a three-electrode setup with Ag/AgCl as the reference electrode and a Pt wire as the counter electrode in a 0.01M Phosphate Buffered Saline (PBS) solution. The measurements were performed either at V_{oc} or at a DC offset potential (doping potential) with an AC amplitude of

0.01V within the frequency range of 0.1Hz – 100KHz. The capacitance-frequency plots for each of the different temperature conditions and doping potentials were calculated using the relation:

$$|Z| = \sqrt{R^2 + X_C^2}$$

where Z is the complex impedance, $X_C$is the imaginary capacitive reactance and R is the real resistance.

$$C = \frac{1}{2\pi f X_C}$$

where C is the capacitance and f is the frequency. The volumetric capacitance was obtained by extracting capacitance values at 0.1Hz from the EIS data followed by division by the product of electrode area and polymer film thickness.

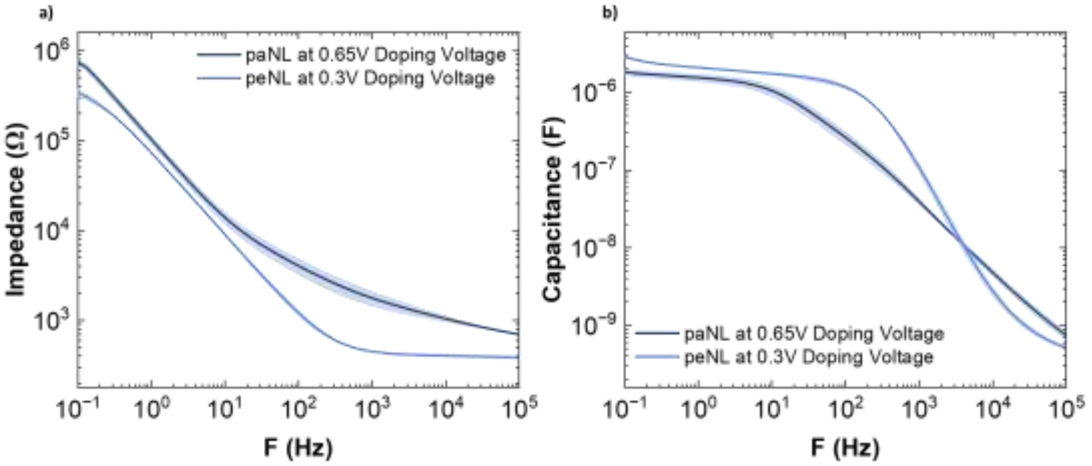

Figure S31. Electrochemical impedance spectroscopy measurements performed on square microfabricated electrodes of a=500 um for the polymers before (dark blue lines) and after (light bluelines) cleavage at 300oC. a) impedance magnitude at the respective doping voltages of maximum transconductance and b) capacitance plots vs frequency for the polymers before and after cleavage at respective doping voltage of maximum. The capacitance values at 0.1 Hz were used to calculate the volumetric capacitance of the polymers.

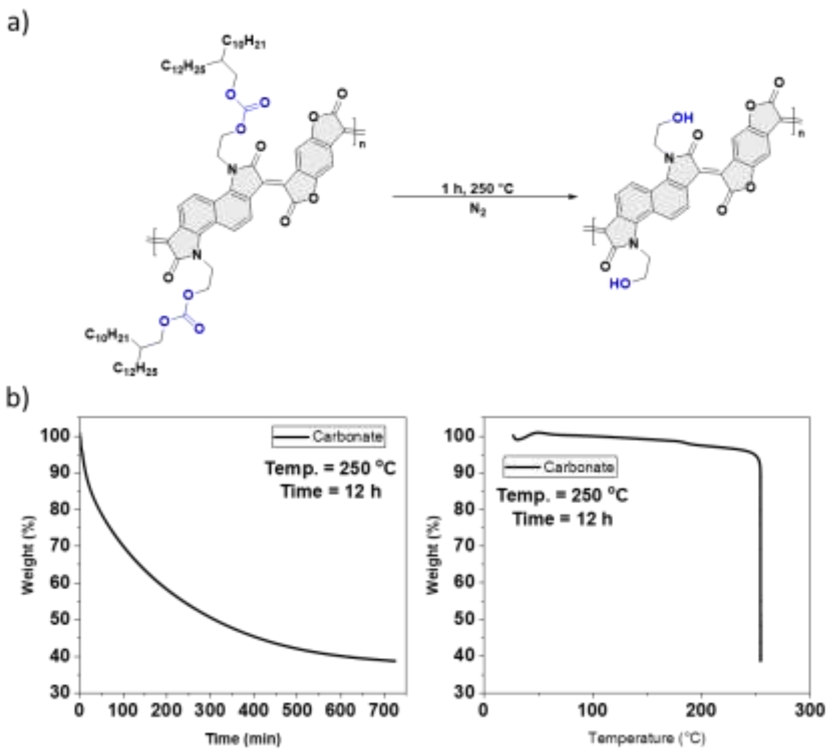

Figure S32. Carbonate rigid rod polymer: a) Chemical structure of the polymer before and after thermal cleavage. b) TGA isothermal at 250 °C for 12 hours.

J. References

1. Emmett, P. H. Gases i n Multimolecular Layers. 407, (1936).
2. Halenda, P. & V, V. " K. 1896, (1951).
3. Singla, P. et al. Multifunctional geometrical isomers of ferrocene-benzo[1,2-b:4,5-b′]difuran-2,6-(3H,7H)-dione adducts: second-order nonlinear optical behaviour and charge transport in thin film OFET devices. J. Mater. Chem. C 5, 697–708 (2017).
4. Alsufyani, M. et al. Lactone Backbone Density in Rigid Electron-Deficient Semiconducting Polymers Enabling High n-type Organic Thermoelectric Performance. Angew. Chemie - Int. Ed. 61, e202113078 (2022).
5. Bernards, D. A. & Malliaras, G. G. Steady-state and transient behavior of organic electrochemical transistors. Adv. Funct. Mater. 17, 3538–3544 (2007).
6. Chen, X. et al. n-Type Rigid Semiconducting Polymers Bearing Oligo(Ethylene Glycol) Side Chains for High-Performance Organic Electrochemical Transistors. Angew. Chemie - Int. Ed. 60, 9368–9373 (2021).
7. Marks, A. et al. Synthetic Nuances to Maximize n-Type Organic Electrochemical Transistor and Thermoelectric Performance in Fused Lactam Polymers. J. Am. Chem. Soc. 144, 4642–4656 (2022).
8. Alsufyani, M. et al. The Effect of Organic Semiconductor Electron Affinity on Preventing Parasitic Oxidation Reactions Limiting Performance of n-Type Organic Electrochemical Transistors. Adv. Mater. 2403911, 1–10 (2024).
9. Surgailis, J. et al. Mixed Conduction in an N-Type Organic Semiconductor in the Absence of Hydrophilic Side-Chains. Adv. Funct. Mater. 31, 2010165 (2021).
10. Sun, H. et al. Complementary Logic Circuits Based on High-Performance n-Type Organic Electrochemical Transistors. Adv. Mater. 30, 1704916 (2018).
11. Wu, H. Y. et al. Influence of Molecular Weight on the Organic Electrochemical Transistor Performance of Ladder-Type Conjugated Polymers. Adv. Mater. 34, 2106235 (2022).
12. Nguyen-Dang, T. et al. Dual-Mode Organic Electrochemical Transistors Based on Self-Doped Conjugated Polyelectrolytes for Reconfigurable Electronics. Adv. Mater. 34, 2200274 (2022).
13. Cho, Y. et al. Small-Molecule Mixed Ionic-Electronic Conductors for Efficient N-Type Electrochemical Transistors: Structure-Function Correlations. Angew. Chemie - Int. Ed. 64, e202414180 (2025).

14. Feng, K. et al. Cyano-Functionalized n-Type Polymer with High Electron Mobility for High-Performance Organic Electrochemical Transistors. Adv. Mater. 34, 2201340 (2022).
15. Tang, H. et al. A solution-processed n-type conducting polymer with ultrahigh conductivity. Nat. 2022 6117935 611, 271–277 (2022).
16. Li, P., Shi, J., Lei, Y., Huang, Z. & Lei, T. Switching p-type to high-performance n-type organic electrochemical transistors via doped state engineering. Nat. Commun. 2022 131 13, 1–8 (2022).
17. Yang, W. et al. High-Performance n-Type Polymeric Mixed Ionic-Electronic Conductors: The Impacts of Halogen Functionalization. Adv. Mater. 36, 2305416 (2024).
18. Ma, M. et al. Regiochemistry and Side-Chain Engineering Enable Efficient N-Type Mixed Conducting Polymers. Angew. Chemie - Int. Ed. 64, e202424820 (2025).
19. Kuang, Y. et al. Matching P- and N-type Organic Electrochemical Transistor Performance Enables a Record High-gain Complementary Inverter. Adv. Mater. 37, 2417691 (2025).
20. Chen, J. et al. Complementary Hydrogen—Bonded Functionalized Mixed Conducting Terpolymers for High-Performance n-type Organic Electrochemical Transistors and Healable Inverters. Angew. Chemie - Int. Ed. 64, e202505011 (2025).
21. Wang, Y. et al. Acceptor Functionalization via Green Chemistry Enables High-Performance n-Type Organic Electrochemical Transistors for Biosensing, Memory Applications. Adv. Funct. Mater. 34, 2304103 (2024).
22. Wu, W. et al. Selenophene Substitution Enabled High-Performance n-Type Polymeric Mixed Ionic-Electronic Conductors for Organic Electrochemical Transistors and Glucose Sensors. Adv. Mater. 36, 2310503 (2024).
